# Atrial Heterogeneity Generates Re-entrant Substrate during Atrial Fibrillation and Anti-arrhythmic Drug Action: Mechanistic Insights from Canine Atrial Models

**DOI:** 10.1371/journal.pcbi.1005245

**Published:** 2016-12-16

**Authors:** Marta Varela, Michael A. Colman, Jules C. Hancox, Oleg V. Aslanidi

**Affiliations:** 1 Department of Biomedical Engineering, Division of Imaging Sciences and Biomedical Engineering, King’s College London, London, United Kingdom; 2 School of Biomedical Sciences, Faculty of Biological Sciences, University of Leeds, Leeds, United Kingdom; 3 School of Physiology, Pharmacology and Neuroscience, Biomedical Sciences Building, University of Bristol, Bristol, United Kingdom; Karlsruhe Institute of Technology KIT, GERMANY

## Abstract

Anti-arrhythmic drug therapy is a frontline treatment for atrial fibrillation (AF), but its success rates are highly variable. This is due to incomplete understanding of the mechanisms of action of specific drugs on the atrial substrate at different stages of AF progression. We aimed to elucidate the role of cellular, tissue and organ level atrial heterogeneities in the generation of a re-entrant substrate during AF progression, and their modulation by the acute action of selected anti-arrhythmic drugs. To explore the complex cell-to-organ mechanisms, a detailed biophysical models of the entire 3D canine atria was developed. The model incorporated atrial geometry and fibre orientation from high-resolution micro-computed tomography, region-specific atrial cell electrophysiology and the effects of progressive AF-induced remodelling. The actions of multi-channel class III anti-arrhythmic agents vernakalant and amiodarone were introduced in the model by inhibiting appropriate ionic channel currents according to experimentally reported concentration-response relationships. AF was initiated by applied ectopic pacing in the pulmonary veins, which led to the generation of localized sustained re-entrant waves (rotors), followed by progressive wave breakdown and rotor multiplication in both atria. The simulated AF scenarios were in agreement with observations in canine models and patients. The 3D atrial simulations revealed that a re-entrant substrate was typically provided by tissue regions of high heterogeneity of action potential duration (APD). Amiodarone increased atrial APD and reduced APD heterogeneity and was more effective in terminating AF than vernakalant, which increased both APD and APD dispersion. In summary, the initiation and sustenance of rotors in AF is linked to atrial APD heterogeneity and APD reduction due to progressive remodelling. Our results suggest that anti-arrhythmic strategies that increase atrial APD without increasing its dispersion are effective in terminating AF.

## Author Summary

The mechanisms behind the most common arrhythmia, atrial fibrillation (AF), remain unclear and anti-arrhythmic drug therapy is often ineffective. In this paper, we develop and apply a novel comprehensive 3D model of canine atria to investigate the role of atrial heterogeneity in the mechanisms of AF and anti-arrhythmic drug action. We find that regions of high heterogeneity of action potential duration (APD) throughout the atria typically provide substrate for arrhythmogenic re-entrant waves during both AF initiation and progression. These mechanistic insights are directly linked with the efficacy of two clinically used class III anti-arrhythmic drugs: amiodarone is more effective at terminating AF than vernakalant, because it leads to an increase in atrial APD without increasing its dispersion. Our computational results are consistent with clinical observations and can help explain the superior efficacy of amiodarone in the clinical treatment of AF at late stages. This framework can easily be extended to investigate the action of other anti-arrhythmic drugs and translated to the human atria. By incorporating patient-specific anatomical and electrophysiological information, and after undergoing careful validation, the proposed *in silico* approach can become a useful tool to evaluate and potentially guide anti-arrhythmic therapy in the clinic.

## Introduction

Atrial fibrillation (AF) is the most common cardiac arrhythmia, imposing a large socio-economic burden on society [1]. Currently, there are approximately 6 million adults in Europe with AF and the number is expected to increase greatly [1]. AF is associated with high morbidity and is often progressive, with electrical and structural remodelling of the atria leading to a substrate that facilitates the self-perpetuation and resistance to treatment of the arrhythmia [1]. The variety of mechanisms of AF onset and progression are incompletely understood [2], which contributes the suboptimal success rates of clinical therapies [3]. Available anti-arrhythmic drugs have major limitations, including poor long-term effectiveness and, for some, high pro-arrhythmic risk [1,3].

Multiple studies have suggested that AF can be sustained by re-entrant waves propagating in an abnormal atrial substrate [2,3]. However, mechanisms for the genesis of these waves during AF remain unclear. Heterogeneous atrial tissue is more susceptible to re-entry leading to conduction block in regions with gradients in refractoriness, high conduction anisotropy, or a combination of these [4]. Experiments have provided evidence that channel blockers aimed at producing anti-arrhythmic effects can instead result in re-entry linked with increased atrial heterogeneity [5].

Moreover, the efficacy of drug therapy for AF is highly variable [1]. Amiodarone is known for its superior efficacy in the clinical treatment of AF at all stages, whereas vernakalant is only indicated for cardioversion of early-onset AF. The multi-channel blocking actions of amiodarone and vernakalant on action potentials (APs) are well-characterised in large animals, particularly the dog. However, mechanistic links between the drug action at the cellular level and the resulting substrate changes at the organ level are unclear and not easily amenable to experimental investigations.

Biophysical modelling has emerged as a powerful tool for dissecting the multi-scale mechanisms of cardiac arrhythmia [6,7] and anti-arrhythmic drugs [8]. In the present study, we developed a detailed biophysical model of 3D canine atria to integrate data from a single species at the cell, tissue and whole atria levels, including multiple effects of remodelling. The dog has been extensively used in experimental AF studies and is arguably the only large species for which information from the ionic channel to the organ level is available for comprehensive validation of the model. We apply it to provide insights into the role of atrial heterogeneity in AF generation and progression and to explore the acute action of amiodarone and vernakalant in the atria *in silico*. We explore the hypothesis that the efficacy of these drugs is linked to their effect on atrial heterogeneity, with the most effective drug actions leading to an overall increase in refractoriness, without increasing its dispersion.

## Methods

### Atrial Single-Cell Models

The Ramirez-Nattel-Courtemanche (RNC) model for the right atrial (RA) cell [9] provides the most widely used description for atrial canine single-cell electrophysiology. However, the importance of the left atrium (LA) and the pulmonary veins (PVs) in AF and the emergence of electrophysiological data from several atrial regions in the dog warrants the development of novel models. Using RNC as the base model and integrating the latest experimental data exclusively from the dog (S3 Table), single-cell models were created for four major atrial regions: RA, LA, PV and Bachmann’s bundle-crista terminalis (BB-CT). The latter two bundles are specialised fast conduction tracts with electrophysiological properties distinctive from the RA, but similar to one another [10,11].

**Fig 1:**
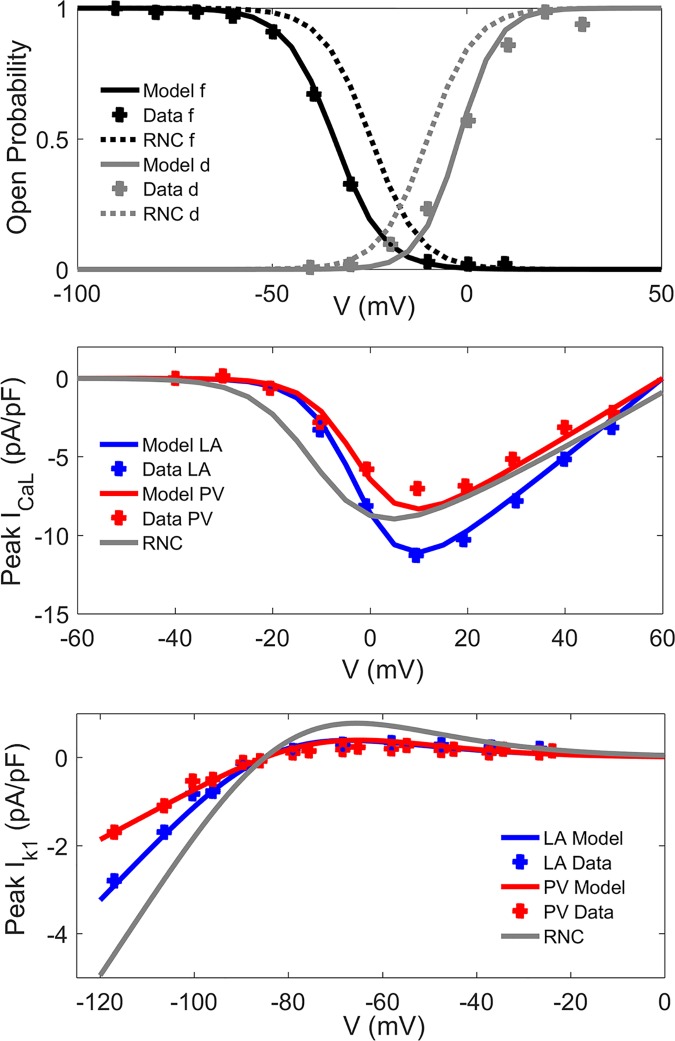
Electrophysiological properties of ionic channel currents in the canine atria. In created atrial single cell models, the properties (lines) are validated against experimental data from the dog (symbols) [11]. **a), b)** L-type calcium current, I_CaL_: **a)** Steady-state values of the voltage-dependent activation (d) and inactivation (f) variables as a function of membrane potential; **b)** Current-voltage relationship in the LA and PV models. **c)** Inward rectifier current, I_K1_: Current-voltage relationships.

**Fig 2:**
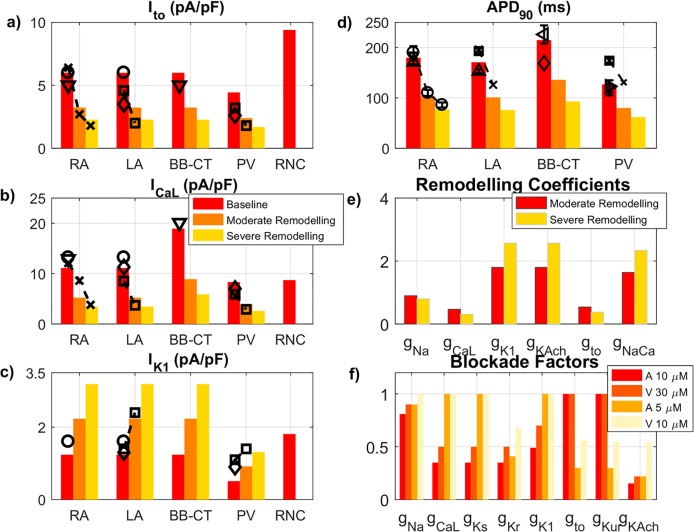
Heterogeneous ionic channel and cellular properties in the canine atria. Created atrial single cell models (bars) are validated against experimental data from the dog (symbols). **a), b), c)** Absolute values of the current densities of three main ionic currents: I_to_ (at +20 mV), I_CaL_ (+10 mV), I_K1_ (-100 mV). Dashed lines join data obtained in the same experimental study. **d)** APD_90_ (1 Hz) for all regional models at different stages of ionic remodelling and the RNC model [9]. **e)** Multiplicative factors for each listed current for models of moderate and advanced ionic remodelling. Only ionic currents that are remodelled with AF are shown. **f)** Blockade factors for amiodarone (A) at concentrations of 5 and 10 μM and vernakalant (V) at 10 and 30 μM for each listed current. Only ionic currents that are affected by the considered drug actions are shown. All sources of experimental data are summarised in S3 and S4 Tables.

Detailed experimental data [10,12–14] were used to update the formulations of major ionic channel currents: I_CaL_ and I_K1_ (Fig 1), I_to_, I_Kur_, I_Kr_ and I_Ks_ (S1–S4 Figs, respectively). A new formulation for the time-dependent hyperpolarization-activated current, I_KACh_, which is constitutively active in the dog [14], was included (S5 Fig). Intracellular calcium handling parameters and regional ionic channel conductances were adjusted to reproduce experimental data (Fig 2, S6 Fig, S1 and S2 Tables). Full details of the models and the complete set of equations can be found in S1 Supporting Text.

Remodelling of ionic channel currents has been well characterised in experimental dog models that develop AF in response to rapid atrial pacing (RAP) [15,16]. Hence, the relevant ionic channel conductances were altered to model the remodelling process in the dog [16–19] at two different stages: moderate (equivalent to 7 days of RAP) and advanced remodelling (42 days of RAP), as shown in Fig 2E and S4 Table. The same ionic remodelling factors were used in all atrial regions [14].

### Atrial Geometry and Fibre Orientation

**Fig 3:**
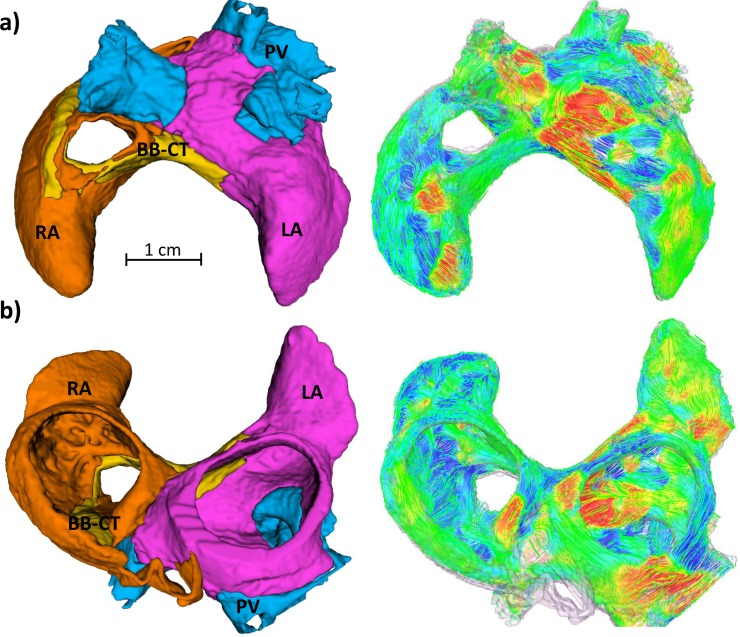
High-resolution 3D canine atrial geometry and corresponding myofibre orientation. **a)** Posterior-superior view; **b)** Inferior-anterior view. The atrial geometry was segmented into four major regions, which are described by the respective electrophysiological atrial cell models: RA (orange), LA (pink), BB-CT (yellow) and PV (blue). Atrial fibres are coloured according to the local fibre orientation component along the anterior-posterior direction.

The canine 3D atrial geometry was obtained through semi-automatic segmentation of 36-μm resolution contrast-enhanced micro computed tomography (microCT) images (Fig 3A) [20]. Details concerning image acquisition and segmentation can be found in previous publications [20,21]. For computational efficiency reasons the image resolution was reduced, producing a computational grid with a spatial step of 300 μm.

Staining of the atria with an iodine solution (I_2_KI) prior to microCT imaging made myofibres hyperintense in relation to surrounding connective tissue in the images [22]. The direction of the myofibres throughout the 3D atria was determined using structure tensor analysis [23]. In short, the direction in which the image intensity changed the least was found by following the eigenvector corresponding to the lowest magnitude eigenvalue of the image's structure tensor. The obtained direction of the myofibres throughout the 3D atria (Fig 3B) was found to be in good agreement with published anatomical descriptions, as described in our previous work [23].

### 3D Biophysical Models

All electrophysiology simulations used the monodomain diffusion-reaction equation, as detailed in the Supporting Text. Anisotropic electrical conduction was incorporated by setting different diffusion coefficients for the directions parallel (D_long_) and perpendicular (D_trans_) to the fibre orientation at each voxel (S5 Table). These were selected to ensure that simulated atrial activation times (AT) and conduction velocities (CV) were in agreement with experimental recordings from canine atria. D_long_ and D_trans_ were reduced by 33–50% in all regions of the atria (“CV reduction” in S5 Table) to mimic the AF-induced structural and gap-junctional remodelling of atrial tissue. We also considered another aspect of structural remodelling, additional to the overall reduction in CV: the increase of the conduction anisotropy ratio (AR = D_long_/D_trans_) due to fibroblast proliferation and collagen deposition along atrial myofibres [24] (“AR Increase” in S5 Table).

Re-entry was initiated in the 3D atrial model by fast pacing in the same site located in the sleeves of the left superior PV (LSPV) using an S_1_-S_2_ protocol, with vulnerable windows computed as described in the Supporting Text. We also characterised re-entrant activity by identifying the number and localization of rotors (re-entrant circuits lasting over 200 ms), stable rotors (lasting over 500 ms) and wavelets (broken activation wavefronts that do not meet the criterion for a rotor) present in the 3D simulations.

### Modelling Anti-Arrhythmic Drug Action

The actions of class III agents amiodarone and vernakalant were modelled as concentration-dependent blockades of relevant ionic channel currents, in accordance with published dose-response curves (Fig 2F, S8 Table). The actions of both drugs were modelled at two different clinically-relevant concentrations also used in canine atrial studies [25]: 10 and 30 μM for vernakalant; 5 and 10 μM for amiodarone. Drug administration was performed after 5 s of self-sustained electrical activity associated with AF and was modelled as an instantaneous and permanent change in macroscopic conductances. In the absence of data on the effect of drugs on remodelled ionic channels, we made the explicit assumption that blockade factors were independent of remodelling.

To gain insights into the mechanisms underlying drug action, we computed: single-cell values of AP duration at 90% repolarisation (APD_90_), 3D atrial APD_90_ and CV magnitude maps and CV restitution curves and effective refractory periods (ERPs) as detailed in the Supporting Text.

## Results

### Model Validation

**Fig 4:**
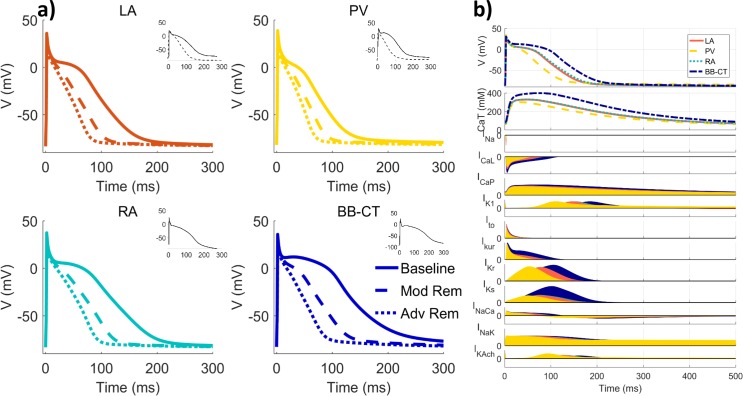
Heterogeneous APs in canine atrial cell models and their changes due to remodelling. **a)** APs (pacing frequency: 2 Hz) for baseline, moderate and advanced ionic remodelling and equivalent canine experimental data when available (insets). **b)** APs for each of the atrial regional models (2 Hz), above intracellular calcium transients (CaT) and each of the models’ currents in relative units. All sources of experimental data are summarised in S3 Table.

The characteristics of the updated ionic channel currents, their heterogeneity and the changes in APs caused by ionic remodelling were in excellent agreement with canine experimental data (Fig 2A–2D and Fig 4). The AP morphologies for all four single-cell models were found to be qualitatively similar to experimental AP recordings from the corresponding atrial regions, as shown in Fig 4. Note that at the borders between tissue types, the APD gradients introduced by the division of atrial tissue into regions with sharp boundaries are greatly smoothed by electrotonic effects (S7 Fig).

Quantitatively, AP properties agreed well with those form experiments (Fig 2D): APD_90_ in the BB-CT cell was the longest (201 ms at 2 Hz), followed by APD_90_ in the RA (166 ms), LA (159 ms) and PV (120 ms). Drug-induced changes in APD_90_ were also in good agreement with experimental data from canine atria (S11 Fig). Both APD_90_ values and APD_90_ dispersion were reduced with increasing degrees of ionic remodelling (Fig 2D and Fig 4). Specifically, APD_90_ at 2 Hz decreases to 60–66% and 45–51% of the baseline values with moderate and advanced ionic remodelling, respectively, in agreement with experimental findings from the dog [14,16]. The maximum upstroke velocity (*dV*/*dt*_max_) was approximately 230 V/s in control conditions, also in good agreement with experiments [25].

The choice of diffusion coefficients resulted in appropriate values of CV and AT in the 3D atria, both in baseline and remodelling conditions, all in excellent agreement with canine data, as shown in S6 and S7 Tables. Thus, simulated CV values were 0.94 and 1.18 m/s in the RA wall and BB-CT bundles, respectively (S6 Table), which is in good agreement with respective experimental data from the dog: 0.8–1.1 m/s in the atrial wall [18,26] and 1.0–1.3 m/s along the BB bundle [27,28]. After remodelling-induced CV reduction (S6 Table), the longitudinal CV in the RA wall was reduced to 0.64 m/s, again in agreement with experimental values of 0.6–0.9 cm/s measured in dogs after RAP [29,30].

### Mechanisms of Arrhythmogenesis

The application of S_1_-S_2_ pacing in the LSPV of the 3D atria model produced arrhythmogenic behaviour in several conditions associated with different stages of AF progression. Table 1 shows the computed vulnerable windows for conduction block and re-entry following such pacing. Conduction block at the PV-LA junction was observed in all conditions, at increasingly shorter values of S_2_ for higher degrees of ionic remodelling. Re-entry around the PVs, on the other hand, was only observed in the presence of both ionic remodelling and CV reduction. An additional increase of AR (linked to fibrotic remodelling) did not considerably affect the computed vulnerable windows. Nevertheless, the complexity of electrical activity increased in these conditions, as additional re-entrant circuits at the RA-CT border quickly appeared, both in moderate and strong ionic remodelling conditions.

**Table 1:** Vulnerable windows for conduction block and re-entry lasting at least 500 ms.

**Conduction Block (ms)**				
**Ionic Remodelling**	**Baseline**	**CV reduction**	**AR increase**	
**None**	155–170	145–165	147–160	
**Moderate**	107–120	85–105	97–117	
**Advanced**	85–97	80–97	75–97	
**Re-Entry (ms)**				
**Ionic Remodelling**	**Baseline**	**CV reduction**		**AR increase**
**None**	−	−		−
**Moderate**	−	93–97		102–105
**Advanced**	−	80–90		92–95

Vulnerable windows change depending on different ionic and structural remodelling conditions.

**Fig 5:**
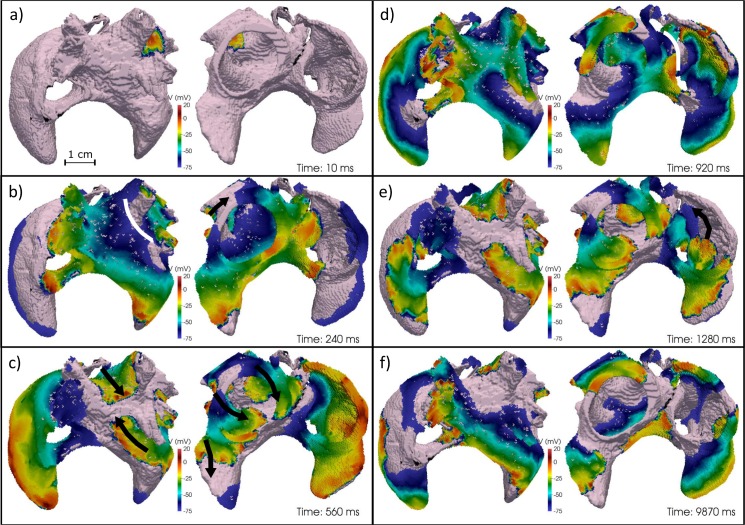
Generation and multiplication of re-entrant waves in the heterogeneous atria. All panels show an anterior-inferior view (left) and a posterior-superior view (right) of the 3D voltage maps at indicated time points. Rotors are represented by black arrows and conduction block by white lines.

Fig 5 shows snapshots of electrical activity in AF, simulated in the conditions of CV reduction and strong ionic remodelling (fixed rate pacing period, S_1_ = 100 ms; period of single extra stimulus, S_2_ = 92 ms)–see S6 Video. Ectopic stimuli in the LSPV (Fig 5A) led to: conduction block (Fig 5B), the subsequent creation of two stable re-entrant circuits around the LSPV and left inferior PV (Fig 5C) and, finally, wave breakdown leading to a secondary rotor in the LA appendage (LAA). As APD was longer in the right side of the atria than in the left, excitation wavefronts emanating from the PVs and spreading into the RA were eventually blocked at the CT (Fig 5D). As a result, a stable rotor was formed in the RA, which was self-sustained and independent of the LA (Fig 5E). Frequent collisions of the RA rotor with wavefronts emanating from the PVs/LA gave the electrical activity in the RA a complex appearance (Fig 5F). This activity was sustained in both atria with a dominant frequency (DF) of 11 Hz for at least 10 s.

With moderate ionic remodelling, a similar activation pattern was observed (S1 Video), except that a stable rotor in the RA was not created. Conduction blocks still emerged due to a combination of APD heterogeneity and anisotropy in the RA, leading to repeated collision of wavelets. DF was 10 Hz in most regions, dropping to 6 Hz in RA regions where wavelet collisions occur.

Intracellular Ca^2+^ dynamics can play an important role in AF mechanisms, primarily by initiating ectopic triggers for AF [2,3], but also by modulating local APD and ERP through Ca^2+^ overload. However, intracellular Ca^2+^ did not substantially affect rotor dynamics in the 3D canine atrial simulations: only insignificant Ca^2+^ accumulation was observed (S8 Fig), in agreement with experimental studies [31].

### Anti-Arrhythmic Drug Action

In all four single-cell models, vernakalant increased the plateau potential and APD_90_ (S9 and S10 Figs) [25,32]. Amiodarone, on the other hand, depressed the plateau (due to the blockade of I_CaL_) and increased the duration of phase 3 (S9 and S10 Figs) [33]. Increasing concentrations of vernakalant and amiodarone gave rise to increases in APD_90_, with 30 μM vernakalant increasing APD_90_ the most (S9 Fig). Intrinsic interregional APD differences were, in general, reduced by amiodarone and increased by vernakalant (part c of S9 Fig). These simulated drug effects are in good agreement with available experimental data (S11 Fig). The action of both drugs reduced *dV*/*dt*_max_ by 30–50% in a concentration-dependent manner [25,32] at the rates associated with AF (6–10 Hz).

In the 3D atrial models, 10 μM amiodarone was very effective at reducing the complexity of the observed electrical activity, eliminating most rotors (Table 2). At 5 μM, amiodarone was less effective, with two rotors remaining in severe remodelling conditions and one stable rotor emerging in the RA in moderate conditions. Vernakalant at all concentrations was in general less effective than amiodarone, causing little change in the observed re-entrant pattern except at high concentrations in advanced remodelling conditions, where two rotors were terminated (Table 2).

**Table 2:** Summary of the electrical activity observed after 5 s in 3D atria.

Ionic Remodelling	Baseline	Vernakalant 10 μM	Vernakalant 30 μM	Amiodarone 5 μM	Amiodaron 10 μM
**Moderate**	**PV:** 2 rotors; **RA:** wavelets; 10.0 Hz	**PV:** 2 rotors; **RA:** wavelets; 8.0 Hz	**PV:** 2 rotors; **RA:** wavelets; 7.4 Hz	**PV:** 2 rotors; **RA:** 1 rotor; 7.7 Hz	Only re-entry in CS
	*(S1 Video)*	*(S2 Video)*	*(S3 Video)*	*(S4 Video)*	*(S5 Video)*
**Advanced**	**PV:** 2 rotors; **LA:** 1 rotor; **RA:** 1 rotor, wavelets; 11.0 Hz	**PV:** 2 rotors; **LA:** 1 rotor; **RA:** 1 rotor, wavelets; 10.0 Hz	**PV:** 1 rotor; **RA:** 1 rotor; **LA:** 1 rotor; 8.3 Hz	**PV:** wavelets; **LA:** 1 rotor; **RA:** 1 rotor; 6.3 Hz	All activity terminated
	*(S6 Video)*	*(S7 Video)*	*(S8 Video)*	*(S9 Video)*	*(S10 Video)*

Electrical activity is simulated at two ionic remodelling stages (including conditions of CV reduction), in baseline (drug-free) conditions and following the application of anti-arrhythmic drugs. The mean dominant frequency in the LA is also shown for each situation when applicable. CS: coronary sinus.

**Fig 6:**
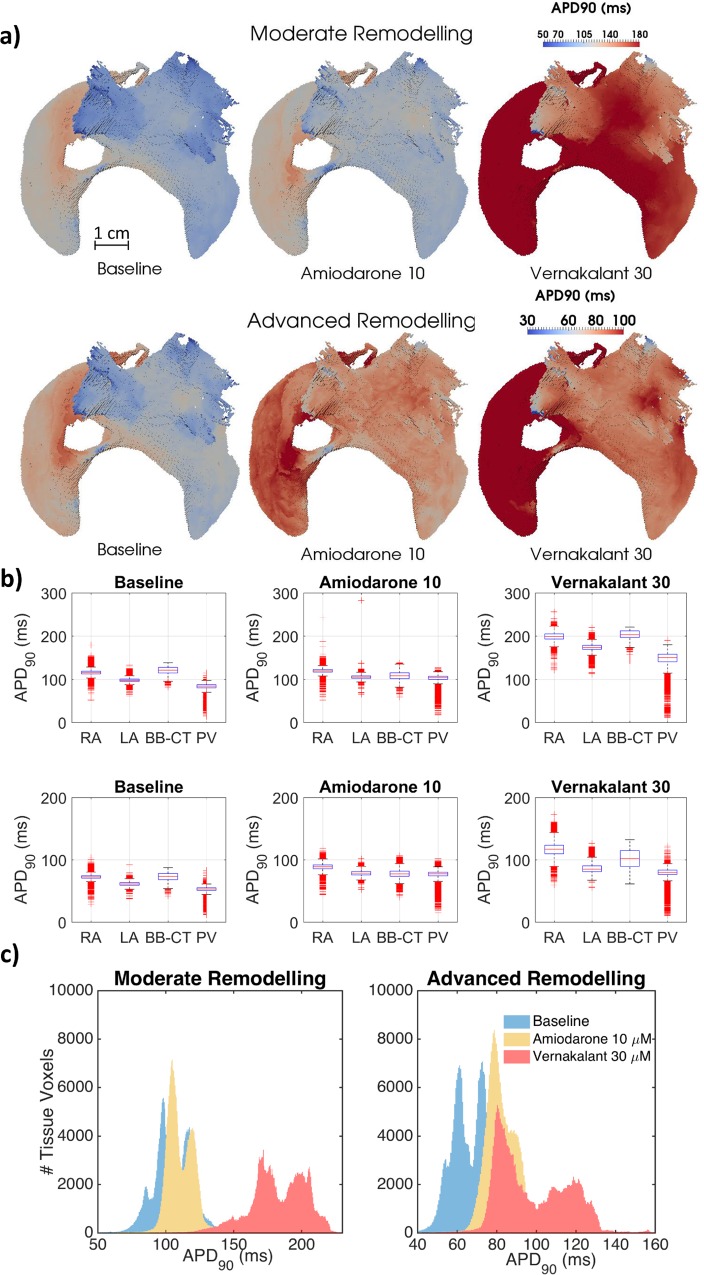
Drug effects on APD_90_ heterogeneity in the 3D canine atrial model. **a)** Whole-atria APD_90_ maps, measured after pacing the atria at 2 Hz from the sinoatrial node at baseline (B) and under the action of amiodarone at 10 μM concentrations and vernakalant at 30 μM. **b)** Boxplots showing mean, standard deviation and spread of APD_90_ measured in each of the major regions of the 3D atria. **c)** Histograms of APD_90_ across the entire atria.

Similar to observations made in single cell simulations (S9 Fig), APD_90_ in the 3D entire atria was increased by both drugs as shown in Fig 6 (and DF were consequently decreased–see Table 2). Fig 6 additionally shows that APD_90_ dispersion in the atria was substantially increased by vernakalant, but reduced by amiodarone. These effects were not only interregional (part c of S9 Fig), but also observed within distinctive atrial regions comprised of the same tissue type (Fig 6A and 6B).

**Fig 7:**
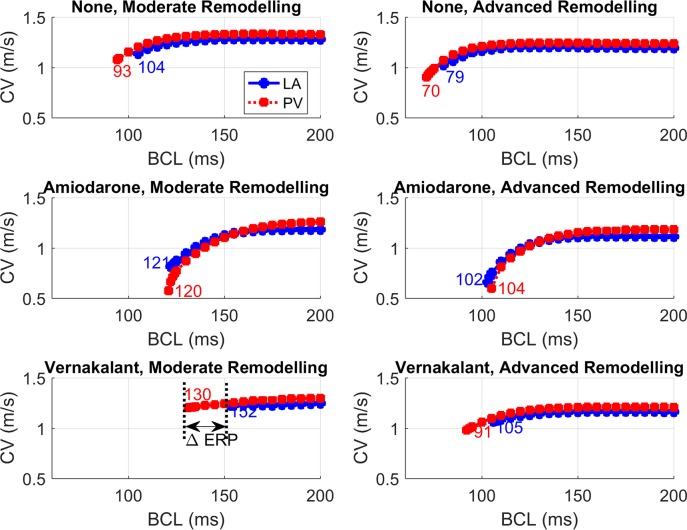
CV restitution curves and ERP calculated with 1D models for the PV and LA. Conditions of moderate (left) and advanced (right) remodelling, at baseline (top) and under the action of 10 μM amiodarone (middle) and 30 μM vernakalant (bottom panels) are shown. The numbers in each panel show ERP in ms (defined as the earliest BCL that does not lead to conduction block) for each tissue type.

**Fig 8:**
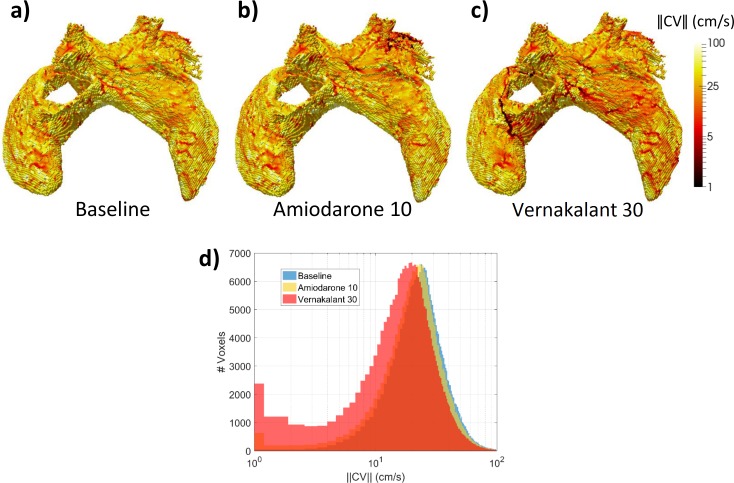
Drug effects on CV heterogeneity in the 3D atria model. Whole-atria CV maps, measured after fast pacing from the LSPV at BCL = 150 ms in moderate remodelling and CV reduction conditions **a)** at baseline; under the actions of: **b)** 10 μM amiodarone and **c)** 30 μM vernakalant. Patchy red regions correspond to zones of decreased CV, which can also be seen in the corresponding histograms in panel **d**), as a slow-conduction left tail, which is most prominent in the presence of vernakalant.

As with APD_90_, amiodarone reduced the dispersion of ERP across different tissue types, whereas vernakalant increased it (Fig 7). The effect of drugs on APD_90_ and EPR dispersion ultimately translates into the dispersion of the magnitude of CV (Fig 8). Due to a partial blockade of I_Na_ by both drugs (Fig 2D, S8 Table), the average CV was decreased throughout the 3D atria (Fig 8). However, only vernakalant led to a higher occurrence of very slow CVs (Fig 8D) and lines of conduction block (Fig 8C), which affected its ability to terminate AF (S13 Fig).

## Discussion

In this study, an electrophysiologically and anatomically detailed model of the canine atria was created and applied to explore the dynamics underlying AF in the entire atria. The model revealed the basic mechanisms of AF development, from ectopic beats in PVs to rotors in regions of high electrical heterogeneity and anisotropy: initially at the PV-LA junctions and, in the advanced stages of AF, also at the RA-CT borders. The interaction of these rotors created multiple wavelets and regions of conduction block, primarily in the RA. The genesis of AF in the model is congruent with patterns seen in experimental and clinical studies [34,35]: a dominant high-frequency "driver" in the PVs, increasingly complex activity in the RA and the emergence of a non-PV driver in chronic AF [34–37]. These are, for the first time, explained by a unified framework that elucidates the mechanisms behind their genesis. Moreover, the model was further applied to explore the acute action of two class III drugs and to propose an explanation for their differential efficacy: actions that increase atrial APD_90_ without increasing APD_90_ dispersion were more successful in terminating AF.

### Biophysical Model Development

Atrial tachypacing in the dog has been extensively used as a model of AF, and applied to investigate its pathophysiological mechanisms, ranging from ionic remodelling [14,16,18] to the relationship between AF and other comorbidities [2]. Furthermore, the effect of multiple anti-arrhythmic drugs in AF has also been studied in detail using canine preparations [25,32,33]. Equally importantly, well-characterised canine atria have similar electrophysiology [9] and anatomy [38] to human atria. Therefore, it is reasonable to propose that an integrative biophysical model of the canine atria, validated by abundant cell-to-organ data from experimental dog models, can improve the understanding of mechanisms of clinical AF and assist in evaluating effects of anti-arrhythmic drugs.

Previous atrial modelling studies often use either 2D shells as geometric models of the atria [39], or assume the 3D atrial myocardium is isotropic [39] or with an anisotropy assigned by descriptive rules [40]. The current study utilises instead a highly-detailed anatomical model for atrial geometry and myofibre orientation, which was created from a high-resolution contrast-enhanced microCT dataset. As structural factors play an important role in wave dynamics (S13 Fig), this model constitutes an important step towards accurately modelling abnormal electrical activity in AF.

In the current study, a novel family of region-specific canine atrial cell models was also developed and validated against experimental data at several levels (Figs 1 and 2, S1–S6 Figs). The update of key ionic currents, such as I_CaL_ and I_K1_, in the RNC model is crucial for simulating the drug action. The resulting models not only accurately reproduced electrical activity in single cells, but also enable validated simulations the spatiotemporal dynamics in the 3D atria in sinus rhythm and AF. This is evidenced by the good agreement of the model with multiple experimental measurements of CV and AT in healthy dogs (S6 and S7 Tables), as well as DF [35,41] in both sinus rhythm and at various AF remodelling stages.

### Genesis of Atrial Fibrillation

This study substantiates a link between re-entrant mechanisms sustaining AF and the underlying heterogeneity and anisotropy of atrial tissue [4,21]. APD heterogeneity [4] and complex myofibre arrangements [21] at the PV-LA junction were responsible for the initial conduction block, which led to the formation of rotors when the wavelength was sufficiently reduced by a combination of ionic remodelling and CV reduction. A similar combination of APD heterogeneity and anisotropy in the border of the RA-CT regions led to further wavebreaks in moderate remodelling conditions and the formation of stable rotors in advanced remodelling (Fig 5). Note that in advanced remodelling secondary rotors also appeared in the LA appendage via a similar mechanism (Fig 5F).

These simulated scenarios of dominant rotors in the PVs and increasingly complex activations in the RA and LA match observations in canine RAP models [41] and clinical studies [35] and are in qualitative agreement with several experimental findings: higher DFs in the LA versus RA [35,41], increased DF with disease/RAP duration [35,41], as well as clinical observations of increasing formation of stable high-frequency sources away from the PVs in persistent compared to paroxysmal AF patients [35].

### Mechanisms of Drug Action

Insights into the role of electrical heterogeneity in AF provided by the 3D atrial model were applied to explore the action of two anti-arrhythmic drugs. In the model, high-concentration amiodarone was found to be more effective at terminating AF than vernakalant at any concentration (Table 2), despite vernakalant’s superior ability to increase APD_90_ and ERP (Figs 6 and 7, S9–S11 Figs). An exception occurs in the CT-RA border where the administration of low-concentration amiodarone led to a small local increase in APD_90_ heterogeneity (Fig 6A) and ultimately to the stabilisation of wavelets into a rotor (Table 2). This can be linked to local high anisotropy (S13 Fig), highlighting the difficulty of predicting drug action in realistic 3D atrial geometries exclusively from single-cell models.

We propose that amiodarone’s high efficacy can be explained by the decrease in the dispersion of refractoriness (measured using either ADP_90_ or ERP) it induces in most regions (Figs 6 and 7). Vernakalant, on the other hand, increases electrical heterogeneity (Fig 6, S9 Fig), leading to CV dispersion and consequently conduction block (Fig 7) and wavebreaks (S13 Fig), similar to a recent canine experimental study with a single-channel blockade [5]. Our findings are in good agreement with amiodarone’s superior clinical effectiveness in the presence of AF remodelling. They also agree with results from previous computational studies performed in a 1D strand using a human cell model, which showed amiodarone to be anti-arrhythmogenic over a wide range of BCLs, for different drug concentrations and in the presence of remodelling [42].

Amiodarone’s ability to reduce APD dispersion can be linked to its blocking effect on I_K1_ and I_CaL_ (Fig 2F), the most heterogeneous currents across the different cell types. This is in contrast to vernakalant, which mostly inhibits outward currents, particularly I_Kur_, which do not change across the atria. As vernakalant does not block I_CaL_, it increases APD to a much greater extent than amiodarone–but it also increases heterogeneity. The studied drug actions cannot however be attributed to the blockade of a single ionic channel. When we considered amiodarone with the ability to block I_K1_ or I_CaL_ removed, or vernakalant with the ability to block I_K1_ or I_CaL_ added, the resulting effects on the CV restitution and ERP dispersion (S12 Fig) supported our initial hypothesis: the most effective drug actions increase ERP but reduce ERP dispersion (S9 and S10 Tables). A similar effect has also been studied in human models of AF, where it was found that regions where AF remodelling promoted a decrease in APD and an increase in APD heterogeneity were most vulnerable to the initiation of re-entry [40].

### Limitations

The presented model does not account for effects of patchy fibrosis [43] or autonomic innervation [44], which will be addressed in detail in future studies. We analysed a simpler condition of increased tissue anisotropy, which can be linked to diffuse interstitial fibrosis. This is representative of both dog models of AF and of 70% of AF patients who do not develop severe patchy fibrosis [45,46]. This modelling approach also enables a separation of purely electrophysiological effects from those related to the presence of patchy fibrosis. Additional patchy fibrosis and vagal innervation can substantially increase electrical heterogeneity in the atria [43,44], potentially contributing to AF through mechanisms similar to those described in the current study.

The action of anti-arrhythmic drugs was modelled as a simple reduction of ionic channel conductances, as is commonly done when modelling the action of class III drugs [8], not taking into account potential rate- and voltage-dependent blocking mechanisms [47]. Despite the care taken in the present study, further detail may need to be included to model more accurately the *in vivo* kinetics of these drugs. However, sufficient experimental data to create such models are currently unavailable and the approach adopted here has effectively highlighted distinctions between the combinations of conductances affected by amiodarone and vernakalant.

## Conclusion

This study demonstrates how *in silico* 3D atrial models can be created from highly detailed ionic channel-to-organ electrophysiological and imaging data and applied to improve the mechanistic understanding of arrhythmogenesis and anti-arrhythmic drug action in AF. The model analyses scenarios of AF onset and progression in the whole atria, from rotor initiation by ectopic beats in the PVs to the emergence of wave breaks and new rotors in both sides of the atria at progressive stages of remodelling. These scenarios are congruent with multiple experimental and clinical observations.

This study demonstrates the dominant role of cell-to-organ atrial heterogeneity in the initiation and sustenance of rotors during AF and the success of anti-arrhythmic drug action in terminating them. To the best of our knowledge, this is the first study where comprehensive whole-atria *in silico* models have been successfully applied to provide a mechanistic link between effects of specific class III drugs at the ionic channel level and the progressive re-entrant substrate for AF in the entire atria.

## Supporting Information

S1 Supporting Text Detailed descriptions of canine single cell atrial models and 3D whole-atria model. (PDF)

S1 Fig Electrophysiological characteristics of the transient outward current, I_to_. **a)** Steady-state values of the activation (o_a_) and inactivation (o_i_) variables as a function of membrane potential. **b)** Time to peak (t_peak_) and inactivation time constant (τ_inac_) as a function of membrane potential. **c)** Peak current-voltage relationship. **d)** Normalised current as a function of time for voltage steps of +50 and +10 mV (from -50 mV) as a function of time. (Experimental data for the LA cell taken from Ehrlich et al., 2003; Li et al., 2001.)
(PNG)

S2 Fig Ultrarapid delayed rectifier current, I_Kur_. Peak current-voltage relationship. (Experimental data (for the LA and RA cells) is taken from Li et al., 2001.)
(PNG)

S3 Fig Rapid delayed rectifier current, I_Kr_. Peak current-voltage relationship. (Experimental data (for the LA and RA cells) is taken from Li et al., 2001.)
(PNG)

S4 Fig Slow delayed rectifier current, I_Ks_. Peak current-voltage relationship. (Experimental data for the LA and RA cells is taken from Ehrlich et al., 2003.)
(PNG)

S5 Fig Time-dependent hyperpolarization-activated current, I_KAch_. **a)** Steady-state values of the voltage-dependent activation variable (x_a_) as a function of membrane potential. **b)** Time constant of the voltage-dependent activation (τ_xa_) variable as a function of membrane potential. **c)** Peak current-voltage relationship. **d)** Normalised current in the LA model as a function of time for voltage steps of -100, -40 and -20 mV (from -40 mV) as a function of time. (All experimental data for both LA and PV cells is taken from Ehrlich et al., 2004.)
(PNG)

S6 Fig Calcium Transient, CaT. CaT in the PV model at 1Hz and comparison with RNC model and experimental data from Coutu et al., 2006.
(PNG)

S7 Fig Effect of electrotonic loading on APD_90_ heterogeneity. APD_90_ was measured across the LA-PV boundary in 3D (full blue line) and compared against single-cell APD_90_ values for corresponding cell types (dashed black line). ERPs (measured in 3D) for each of the tissue types are also shown (dark blue circles). Simulations were carried out at a BCL of 300 ms, with moderate ionic remodelling and CV reduction conditions.
(PNG)

S8 Fig Temporal evolution of the intracellular concentration of Ca^2+^ (Cai) in the 3D model after fast pacing. Pacing was performed in the left superior PV, in conditions of CV reduction and moderate ionic remodelling. Impulses are applied at 0 and 200 ms. The depolarizing wavefront (V > - 20mV) can be seen in red on the right hand side of each panel for the same time points as the Cai maps on the left hand side. Time since initial pacing: **a)** 10 ms, **b)** 100 ms, **c)** 210 ms, **d)** 300 ms.
(PNG)

S9 Fig Effect of anti-arrhythmic drugs on single cell action potentials. **a)** Action potentials for the right atrium, paced at 2 Hz, in the absence of drugs and after administration of vernakalant or amiodarone for different degrees of ionic remodelling. **b)** Absolute APD_90_ values in all cell types and **c)** differences in APD_90_ between right atrial tissues (BB-CT and RA) and left atrial tissues (PV and LA) for: different degrees of remodelling (none, moderate or advanced) at baseline (B) and after the application of either 5, 10 μM of amiodarone (A5, A10) or 10, 30 μM of vernakalant (V10, V30).
(PNG)

S10 Fig APD_90_ restitution curves. APD_90_ restitution curves in baseline (full line), moderate (dashed line) and advanced (dotted line) ionic remodelling for all atrial cell types in baseline conditions (top panel) and after the administration of 30 μM of vernakalant (centre panel) or 10 μM amiodarone (bottom panel).
(PNG)

S11 Fig Relative changes in ERP and APD_90_ induced by drug administration. Experimental data (black) is taken from Sicouri et al., 2012 for the action of vernakalant 30 μM on healthy canine PVs and Sicouri et al., 2010 for the action of chronic amiodarone on healthy canine RA cells. Simulations were carried out for the PV and LA cells using the described 1D cable model (for ERP calculations) and single-cell models (for APD_90_) at a BCL of 500 ms in the absence of any remodelling.
(PNG)

S12 Fig Conduction velocity restitution curves. Conduction velocity restitution curves simulated in 1D for PV (red) and LA (blue) cells, in conditions of moderate (full lines) and severe remodelling (dashed lines). Panels show CV restitution curves for baseline values (top), in the presence of amiodarone 10 μM (a), second panel) and vernakalant 30 μM (b), second panel). The bottom two panels depict CV restitution curves for single-ionic channel modifications of amiodarone 10 μM (a)) or vernakalant 30 μM (b)).
(PNG)

S13 Fig Atrial activation times (ATs) in the presence of drugs in the 3D canine model. Superior-posterior view of the atria, showing ATs during AF (moderate ionic remodelling, CV reduction conditions) at a) baseline and after the application of b) amiodarone 10 μM and c) vernakalant 30 μM. Panel d) shows the 3D fibre orientation in the same geometry. The rotor around the LSPV (arrow) is terminated near the PVs by amiodarone (dashed lines), whereas vernakalant creates an additional conduction block at the CT (full line).
(PNG)

S1 Table Conductances of the currents used in each regional model. *The formulation of these currents is also cell-type dependent—see the last chapter of S1 Supporting Text for details.
(PDF)

S2 Table Ca^2+^-handling variables in the proposed family of models and the RNC model. Only parameters whose values differ in the two models are listed.
(PDF)

S3 Table Literature references for the canine experimental data shown in Figs 1, 3 and 4 in the manuscript. (PDF)

S4 Table Multiplicative factors for ionic remodelling of each current. These are shown in conditions of moderate (7-days of RAP) and severe (42-days of RAP) remodelling, accompanied by the relevant references.
(PDF)

S5 Table Values for the longitudinal diffusion coefficient (D_long_) and anisotropy ratio (AR) for each region of the atria. These are shown for three different stages of structural remodelling: baseline, CV reduction and additional AR increase.
(PDF)

S6 Table Longitudinal conduction velocity values (CV_longitudinal_) measured in the 3D atrial model. Values from the current study computed at a BCL of 350 ms are shown alongside corresponding experimental values measured experimentally in dogs.
(PDF)

S7 Table Activation times in different regions when pacing from the sinoatrial node at a BCL of 350 ms. SVC: superior vena cava; RAA: right atrial appendage; IVC: inferior vena cava; LBB: left portion of Bachmann's bundle; LAA: left atrial appendage; RSPV: right superior pulmonary vein; RIPV: right inferior pulmonary vein.
(PDF)

S8 Table Blockade factors for amiodarone and vernakalant. The values used to model 5 and 10 μM amiodarone (Amio) and 10 and 30 μM vernakalant (Verna) are accompanied by IC_50_ values from the literature for each current affected by the drug. Corresponding literature references are also shown.
(PDF)

S9 Table Outcome of 3D simulations after the administration of modified drug actions. Modified amiodarone 10 μM (Amio 10) and 30 μM vernakalant (Verna 30) drugs were applied and their outcome is compared to baseline (no drug) conditions. All simulations lasted 5 s. Blockade factors for additional I_K1_ and I_CaL_ blocks were 0.60.
(PDF)

S10 Table Relationship between the effective refractory period (ERP), its dispersion and the outcome of 3D simulations in the presence of the analysed drug actions. We show the changes in effective refractory period (ERP) and dispersion of ERP between the LA and PV cells effected by the analysed drug actions (shown in S12 Fig), as well as the qualitative outcome of 3D simulations (S9 Table) for the same drug actions.
(PDF)

S1 Video Spatiotemporal dynamics of electrical activity in the 3D atria in baseline (no drugs), CV reduction and moderate ionic remodelling conditions. (MP4)

S2 Video Spatiotemporal dynamics of electrical activity in the 3D atria after the administration of vernakalant (10 μM), in CV reduction and moderate ionic remodelling conditions. (MP4)

S3 Video Spatiotemporal dynamics of electrical activity in the 3D atria after the administration of vernakalant (30 μM), in CV reduction and moderate ionic remodelling conditions. (MP4)

S4 Video Spatiotemporal dynamics of electrical activity in the 3D atria after the administration of amiodarone (5 μM), in CV reduction and moderate ionic remodelling conditions. (MP4)

S5 Video Spatiotemporal dynamics of electrical activity in the 3D atria after the administration of amiodarone (10 μM), in CV reduction and moderate ionic remodelling conditions. (MP4)

S6 Video Spatiotemporal dynamics of electrical activity in the 3D atria in baseline (no drugs), CV reduction and advanced remodelling conditions. (MP4)

S7 Video Spatiotemporal dynamics of electrical activity in the 3D atria after the administration of vernakalant (10 μM), in CV reduction and advanced remodelling conditions. (MP4)

S8 Video Spatiotemporal dynamics of electrical activity in the 3D atria after the administration of vernakalant (30 μM), in CV reduction and advanced remodelling conditions. (MP4)

S9 Video Spatiotemporal dynamics of electrical activity in the 3D atria after the administration of amiodarone (5 μM), in CV reduction and advanced remodelling conditions. (MP4)

S10 Video Spatiotemporal dynamics of electrical activity in the 3D atria after the administration of amiodarone (10 μM), in CV reduction and advanced remodelling conditions. (MP4)
