# Supplementary material for: Atrial Heterogeneity Generates Re-entrant Substrate during Atrial Fibrillation and Anti-arrhythmic Drug Action: Mechanistic Insights from Canine Atrial Models
Source: PLoS Comput Biol. 2016 Dec 16;12(12):e1005245. doi: 10.1371/journal.pcbi.1005245 (PMC5161306; doi:10.1371/journal.pcbi.1005245)
Supplement: S1 Supporting Text — (PDF) [file pcbi.1005245.s001.pdf]

Online Supplement - Atrial heterogeneity generates reentrant substrate during atrial fibrillation and anti-arrhythmic drug action: mechanistic insights from canine atrial models

M. Varela, M. A. Colman, J. C. Hancox, O. V. Aslanidi

Contents

1 Single-Cell Models **2**
1.1 Transient Outward Current, I_{to} 2
1.2 Ultrarapid Delayed Rectifier Current, I_{Kur} 3
1.3 Rapid Delayed Rectifier Current, I_{Kr} 4
1.4 Slow Delayed Rectifier Current, I_{Ks} 5
1.5 Inward Rectifier Current, I_{K1} 5
1.6 L-Type Calcium Current, I_{CaL} 5
1.7 Time-Dependent Hyperpolarization-Activated Current, I_{KACh} 6
1.8 Intracellular Ca^{2+} Handling 7
1.9 Other Currents 8
1.10 Literature References for Figures in Manuscript 9

2 Details of the Biophysical Model **10**
2.1 Simulation Parameters 10
2.2 Structural Remodelling Parameters 11
2.3 Computation of Vulnerable Windows 13
2.4 Intracellular Ca^{2+} in the 3D Model 13
2.5 Modelling Anti-Arrhythmic Drug Action 14
2.6 Conduction Velocity, APD_{90} and Effective Refractory Period Measurements 18
2.7 Role of I_{K1} and I_{CaL} in Drug Action 19
2.8 Atrial Activation Maps under Drug Action 21

3 Legends of Supplementary Videos **22**

4 Mathematical Formulation of Ionic Currents **26**
4.1 I_{to} 27
4.2 I_{Kur} 27
4.3 I_{Kr} 27
4.4 I_{Ks} 28
4.5 I_{K1} 28
4.6 I_{CaL} 28
4.7 I_{KAch} 28

Abbreviations

AP	Action Potential
APD_{90}	90% Action Potential Duration
AT	Activation Time
BB	Bachmann's (Anterior Interatrial) Bundle
Cai	Intracellular Ca^{2+} Concentration
CaT	Calcium Transient
CT	Crista Terminalis
CV	Conduction Velocity
ERP	Effective Refractory Period
I_{K1}	K^+ Inward Rectifier Current
I_{KAch}	K^+ Time-Dependent Hyperpolarization-Activated Current
I_{Kr}	K^+ Rapid Delayed Rectifier Current
I_{Ks}	K^+ Slow Delayed Rectifier Current
I_{Kur}	K^+ Ultrarapid Delayed Rectifier Current
I_{to}	K^+ Transient Outward Current
I_{Na}	Fast Na^+ Current
I_{NaCa}	Na^+-Ca^{2+} Exchanger Current
I_{NaK}	Na^+-K^+ Exchanger Current
I_{CaL}	L-Type Ca^{2+} Current
I_{CaP}	Ca^{2+} Pump Current
I_{bCa}	Background Ca^{2+} Current
I_{bNa}	Background Na^+ Current
I_{bCl}	Background Cl^- Current
IC_{50}	Half-Maximal Inhibitory Concentration
LA	Left Atrium
PV	Pulmonary Veins
RA	Right Atrium
RNC	Ramirez-Nattel-Courtemanche
SR	Sarcoplasmic Reticulum
VW	Vulnerable Window

1 Single-Cell Models

1.1 Transient Outward Current, I_{to}

The steady-state voltage-dependent activation and inactivation curves and time constants for I_{to} were all fitted to experimental data from canine LA and PV cells [1]. Both the activation and inactivation curves were shifted by -12.5 mV relative to the RNC model (**Figure S1a)**). The activation time constant was increased and the inactivation time constant, decreased (to, respectively, 1.5 and 14.2 ms at +20 mV, compared with 4 and 9.5 ms in the RNC model) (**Figure S1b)**). The maximum conductance, g_{to}, was determined for the LA cell model by fitting the simulated current-voltage curves for I_{to} to the respective experimental data (**Figure S1c)**), ensuring that the simulated time course of I_{to} also matched the respective experimental recordings (**Figure S1d)**). The formulation of I_{to} in the LA model was also used in the RA and BB-CT models, as experiments show no significant change in I_{to} between these regions in the canine atria

[2, 3]. For the PV model, the value of g_{to} was reduced by a factor of 0.74 to match experimental findings [1], as stated in **Table S1**.

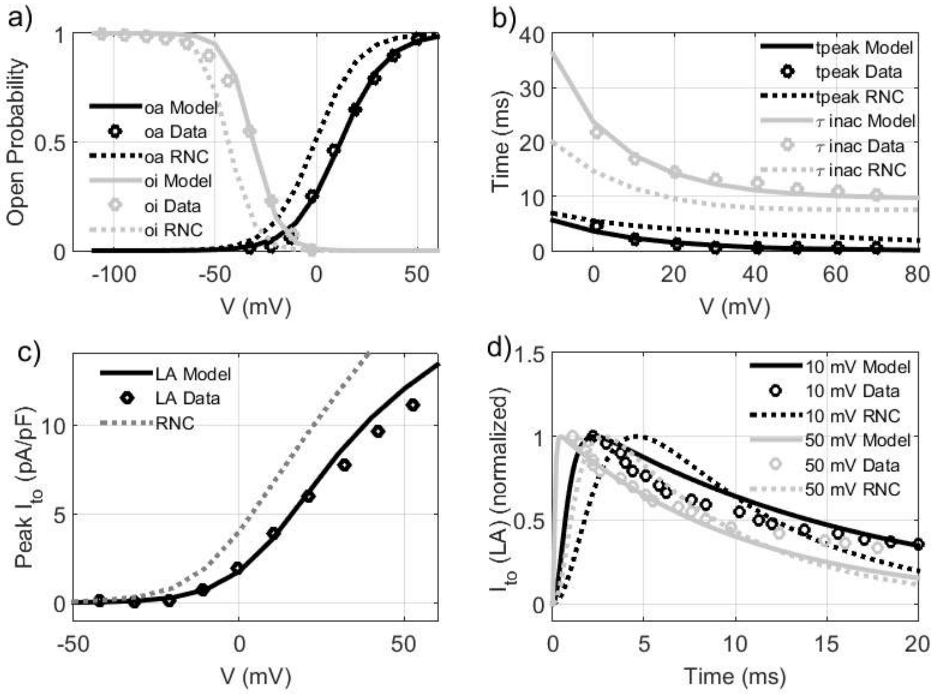

Figure S 1: Electrophysiological characteristics of the transient outward current, I_{to}: a) Steady-state values of the activation (o_a) and inactivation (o_i) variables as a function of membrane potential. **b)** Time to peak (t_{peak}) and inactivation time constant (τ_{inac}) as a function of membrane potential. **c)** Peak current-voltage relationship. **d)** Normalised current as a function of time for voltage steps of +50 and +10 mV (from -50 mV) as a function of time. (Experimental data for the LA cell taken from [1, 2].)

1.2 Ultrarapid Delayed Rectifier Current, I_{Kur}

The formulation of I_{Kur} in the RNC model was updated based on experimental data from canine LA cells [2]. The voltage-dependent conductance, $g_{Kur} * f_{Kur}(V)$, was split into two parts and refitted such that the resultant model produced experimentally validated current densities (**Figure S2**). The simulated current density of I_{Kur} was 5.8 pA/pF at +40 mV , compared to 11.3 pA/pF in the RNC model. The formulation of I_{Kur} in the LA model was also used in the PV, RA and BB-CT models, as experiments show no significant change in I_{Kur} between these regions in the canine atria [2, 3].

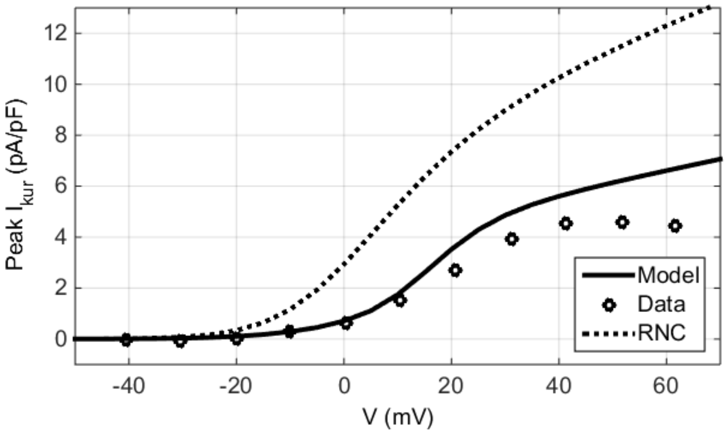

Figure S 2: Ultrarapid delayed rectifier current, I_{Kur}**:** Peak current-voltage relationship. Experimental data (for the LA and RA cells) is taken from [2].

1.3 Rapid Delayed Rectifier Current, I_{Kr}

The formulation of I_{Kr} in the RNC model was updated based on experimental data from the canine LA and RA cells [2]. The voltage-dependent conductance, $g_{kr} * f_{kr}(V)$, was refitted such that the resultant LA and RA models produced experimentally validated current densities (**Figure S3**). Compared to the RNC model (1.0 pA/pF at +40 mV), this results in higher current densities in the LA and RA cell models (respectively, 1.7 and 1.1 pA/pF) (**Figure S3**). The PV model follows that of the LA with a conductance 1.55-fold higher, to match the experimentally seen difference in the current density of I_{Kr} between the PV and LA cells [1]. The BB-CT model uses the same I_{Kr} formulation as the RA [3].

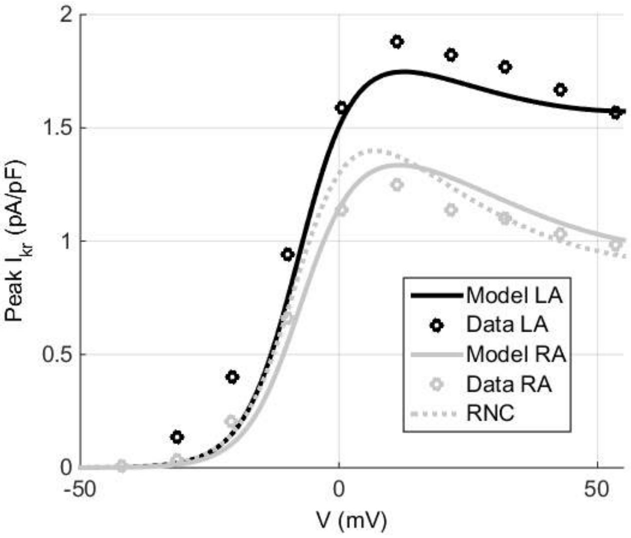

Figure S 3: Rapid delayed rectifier current, I_{Kr}**:** Peak current-voltage relationship. Experimental data (for the LA and RA cells) is taken from [2].

1.4 Slow Delayed Rectifier Current, I_{Ks}

The simulated current-voltage curves for I_{Ks} were fitted to the respective experimental data [1] to adjust the maximal conductance (**Figure S4**). Relative to the RNC model (6.5 pA/pF at +40 mV), the simulated current density in the LA cell model was slightly decreased and, in the PV model, increased: 6 and 9.5 pA/pF, respectively. The formulation of I_{Ks} in the LA model was also used in the RA and BB-CT models, as experiments in canine models report no intra-atrial differences in I_{Ks} [2, 3].

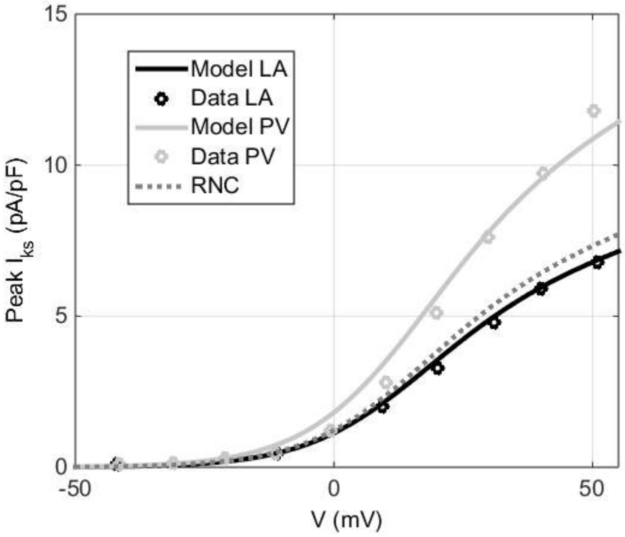

Figure S 4: Slow delayed rectifier current, I_{Ks}: Peak current-voltage relationship. Experimental data for the LA and RA cells is taken from [1].

1.5 Inward Rectifier Current, I_{K1}

The formulation of I_{K1} in the RNC model was modified based on experimental data from the canine LA and PV cells [1]. The simulated current densities in the resultant LA and PV models were relatively low: -1.1 and -0.8 pA/pF at -100 mV, respectively, compared to -1.8 pA/pF in the RNC model. The formulation of I_{K1} in the LA model was also used in the RA and BB-CT models, as experiments report no intra-atrial differences in I_{K1} [2, 3]. No differences in I_{K1} have been observed in patients either in sinus rhythm or chronic AF [4, 5]. Therefore, we have not included a left-to-right gradient in I_{K1} in this study of canine atria model.

1.6 L-Type Calcium Current, I_{CaL}

The formulation of I_{CaL} in the RNC model was modified based on experimental data from the canine LA and PV cells [1]. The steady-state voltage-dependent activation curve, $d_\infty(V)$, and the steady-state voltage-dependent inactivation curve, $f_\infty(V)$, were shifted by -8 mV and +10 mV, respectively. This results in a smaller current activation window. The maximum conductance, g_{CaL}, was determined for the LA and PV cell models by fitting the simulated current-voltage curves to the respective experimental data [1]. The resultant value of g_{CaL} in the LA model was 1.33-fold higher than that in the PV models [1], as shown in **Table S1**. g_{CaL} in the RA model was set to the same value as in the LA model, since experiments report no difference in the current density of

I_{CaL} between RA and LA cells [2]. The value of g_{CaL} in the BB-CT model was increased 1.71 times relative to the LA and RA models, to match the observed difference in the current density of I_{CaL} between the BB-CT and RA [3].

1.7 Time-Dependent Hyperpolarization-Activated Current, I_{KACh}

Our model includes the K^+ time-dependent hyperpolarization-activated current carried by Kir3 subunits that has been found to be constitutively active in the dog [6, 7] and which is not included in the RNC model. This current will be referred to as I_{KAch}, as it has been suggested it has the same structure and properties as the acetylcholine-activated current, but does not require agonist binding for activation [8].

The formulation of I_{KAch} includes the voltage-dependent conductance, $g_{KAch}*f_{KAch}(V)$, and voltage- and time-dependent activation variable, x_a, all of which were described based on experimental data from dog LA cells [6] (**Figure S5a)** and **b**). The same model was used for all cell types, with g_{KAch} in the PV model 1.56 times higher than that of the LA cell, to match the experimentally seen differences in the current density of I_{KAch} [6] (**Figure S5c)**). The value of g_{KAch} for the LA was used in the RA and BB-CT models, due to the lack of experimental data from the dog on intra-atrial differences in this current.

Acetylcholine-mediated I_{KACh} gradients can develop in the atria in conditions of increased vagal tone [5]. Vagal effects, including the emergence of additional acetylcholine-mediated gradients of refractoriness, were beyond the scope of the present study, which focused on electrophysiological heterogeneities that are not directly mediated by agonists.

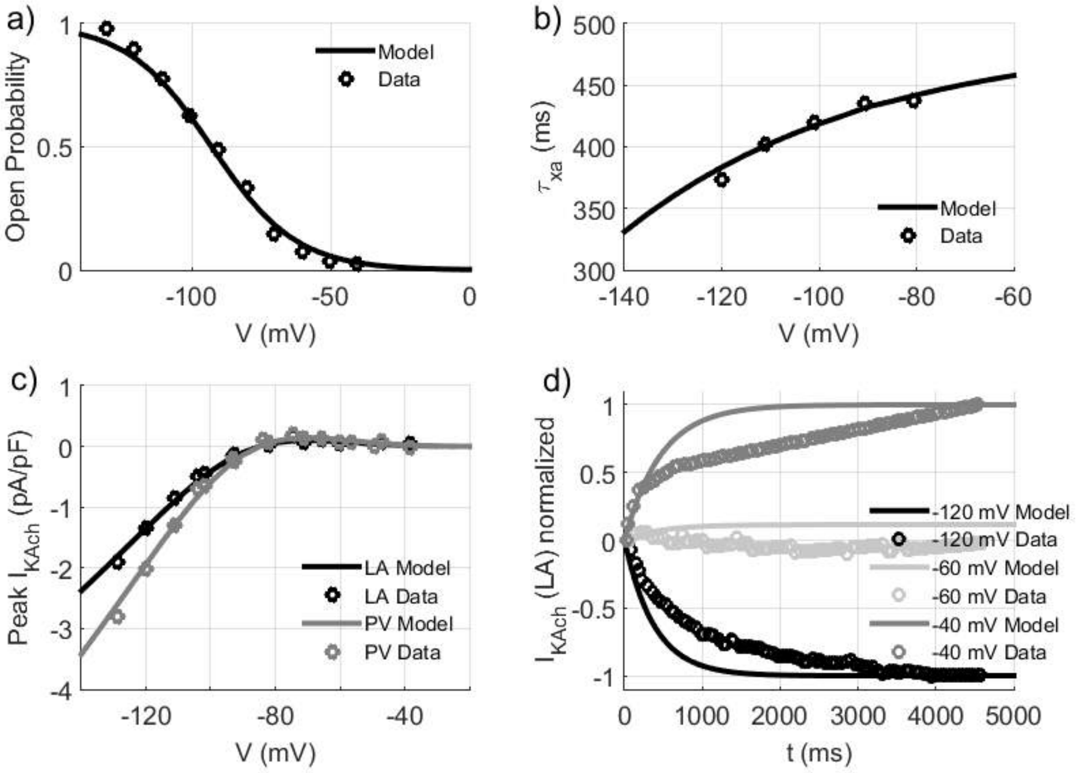

Figure S 5: Time-dependent hyperpolarization-activated current, I_{KAch}: a) Steady-state values of the voltage-dependent activation variable (x_a) as a function of membrane potential. **b)** Time constant of the voltage-dependent activation (τ_{xa}) variable as a function of membrane potential. **c)** Peak current-voltage relationship. **d)** Normalised current in the LA model as a function of time for voltage steps of -100, -40 and -20 mV (from -40 mV) as a function of time. (All experimental data for both LA and PV cells taken from [6].)

1.8 Intracellular Ca^{2+} Handling

We used the same formulation for the intracellular Ca^{2+} handling as the RNC model. However, model parameters were updated such that the model produced Ca^{2+} transients similar to those recorded experimentally [9, 10]. Specific updates included: a 1.5-fold decrease in the maximal Ca^{2+} release rate from the sarcoplasmic reticulum (SR), g_{rel}; a reduction by a factor of 0.65 in the half-saturation constant for Ca^{2+} uptake into the SR, k_{up}; a reduction by a factor of 0.7 in the maximal Ca^{2+} uptake rate; an increase (by a factor of 1.8) in the maximal Ca^{2+} concentration in the uptake compartment of the SR and finally a 1.4-fold increase in the time constant of the activation gate (u) of the Ca^{2+} release current from the SR. All these parameters are listed in **Table S2**.

The same description of Ca^{2+} handling was used in the LA, RA, PV and BB-CT cell models, as experimental studies have not reported any significant differences in Ca^{2+} transients and Ca^{2+} handling parameters between cells from various regions of the canine atria [10]. The resulting calcium transient amplitudes, CaT (peak: 350 nM, at 2 Hz), are lower than in the RNC model (peak: 705 nM), in good agreement with experiments

performed in canine atria [9, 10] - see **Figure S6**.

Remodelling led to a reduction in the amplitude of CaT (220 nM) compared to values in non-remodelled conditions (350 nM) paced at the same physiological rate (2 Hz). These modelling results are in agreement with experiments that have reported a reduction in CaT in dogs following rapid atrial pacing [9, 10]. The reduction of CaT and the absence of intracellular Ca^{2+} overload in our models is also in agreement with recent experimental results reporting silencing of subcellular Ca^{2+} signalling in atrial myocytes due to remodelling [11].

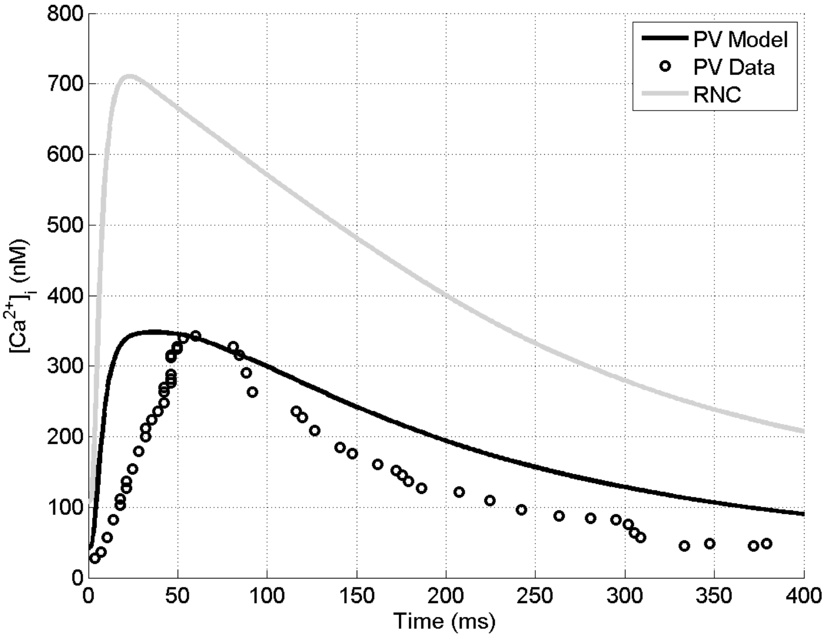

Figure S 6: Calcium Transient, CaT. CaT in the PV model at 1Hz and comparison with RNC model and experimental data from [10].

1.9 Other Currents

There are no experimental reports of regional heterogeneity of the following currents in the canine atria: the Ca^{2+} pump current, I_{CaP}, $Na^{+} - K^{+}$ exchange current, I_{NaK}, Na^{+}-Ca^{2+} exchange current, I_{NaCa} and the fast Na^{+} current, I_{Na} for all cell types. We therefore kept the RNC formulations of these currents for all cell types. The conductances of small Na^{+}, Ca^{2+} and Cl^{-} background currents, I_{bNa}, and I_{bCa} , were adjusted such that the models produced stable resting membrane potentials and balanced ionic concentrations. The Ca^{2+}-activated Cl^{-} current, which is present in the RNC model but whose contribution to the action potential was found to be negligible, was removed.

The maximal conductances of all the currents used in the four regional models are listed in **Table S1**. The mathematical formulation for all the modified currents is in Chapter 3. All other currents were not changed relative to the RNC model [12].

Conductance (nS/pF)	PV	LA	RA	BB - CT	RNC
**gNa**	7.8	7.8	7.8	7.8	7.8
**gCaL**	0.255	0.34	0.34	0.58	0.24
**gCaP**	0.275	0.275	0.275	0.275	0.275
**gK1***	0.036	0.1	0.1	0.1	0.15
**gto**	0.07104	0.096	0.096	0.096	0.19824
**gKur**	0.0115	0.0115	0.0115	0.0115	1
**gbCl**	0.0055	0.0008	0.0008	0.0008	0
**gKr***	0.022475	0.0145	0.00899	0.00899	0.06984
**gKs**	0.0832	0.052	0.052	0.052	0.0561
**gbNa**	1.00E-05	1.00E-05	1.00E-05	1.00E-05	6.74E-04
**gbCa**	1.00E-05	1.00E-05	1.00E-05	1.00E-05	1.13E-03
**gNaCa**	1600	1600	1600	1600	1600
**gNaK**	0.6	0.6	0.6	0.6	0.6
**gKAch**	0.0065	0.0045	0.0045	0.0045	0

Table S 1: Conductances of the currents used in each regional model. * The formulation of these currents is also cell-type dependent - see Chapter 4 for details.

Variable	RNC Model	RA, LA, BB-CT, PV	Units
k_{up}	9.2×10^{-4}	6.0×10^{-4}	μM
$i_{up,max}$	5.0×10^{-3}	3.5×10^{-3}	mM/ms
$ca_{up,max}$	15.0	27.0	mM
g_{rel}	30.0	8.0	kHz
τ_u	8.0	11.2	ms

Table S 2: Ca^{2+}-handling variables in the proposed family of models and the RNC model. Only parameters whose values differ in the two models are listed.

1.10 Literature References for Figures in Manuscript

The sources of experimental data used in **Figures 1** and **3** in the manuscript are referenced in **Tables S3** and S4.

Source of canine experimental data	Symbol in Figure 1a-c	Symbol in Figure 1d	AP data in Figure 3
**Ehrlich et al, 2003 [1]**	$\Diamond$	$\triangle$	
**Li et al, 2001 [2]**	O	$\square$	LA (panel a)
**Cha et al, 2005 [7]**	$\square$	X	LA, PV (panels a,b)
**Yue et al, 1997 [13]**	X	O	
**Burashnikov et al, 2004 [14]**		$<$	RA, BB-CT (panels c,d)
**Chen et al, 2006 [15]**		$>$	

Table S 3: Literature references for the canine experimental data shown in Figures 1, 3 and 4 in the manuscript.

Current	Moderate Remodelling	Severe Remodelling	References
I_{Na}	0.90	0.80	[16]
I_{CaL}	0.47	0.31	[13, 7]
I_{K1}	1.80	2.57	[7]
I_{KAch}	0.54	0.38	[8]
I_{to}	1.64	2.34	[13, 7]
I_{NaCa}	1.8	2.57	[17]

Table S 4: Multiplicative factors for ionic remodelling of each current. These are shown in conditions of moderate (7-days of RAP) and severe (42-days of RAP) remodelling, accompanied by the relevant references.

2 Details of the Biophysical Model

2.1 Simulation Parameters

All electrophysiological simulations used the monodomain diffusion-reaction equation:

$$\frac{\partial V_m}{\partial t} = \nabla . D \nabla V_m - \frac{I_{ion}}{C_m} \quad (1)$$

where V_m is the transmembrane potential, t is time, C_m is the membrane capacitance, I_{ion} is the transmembrane ionic current per unit area, D is the diffusion (normalised conductivity) tensor. It was solved using a centred finite differences method in a rectangular grid with 180x110x185 voxels and a forward Euler method with a time step of 2.5 μs, a spatial step of 0.3 mm and zero-flux boundary conditions. These parameters were found to lead to stable solutions over the explored range of conditions and simulation lengths. All simulations were run on 80 CPUs in a SGI UV 1000 high-performance computer, using custom-written C code parallelised using MPI. Under these conditions, wall clock time for 1s of simulation was approximately 4h15min. All data visualisation and post-processing was performed using Paraview (Kitware, Clifton Park, NY, USA) and Matlab (Mathworks, Natick MA, USA).

Although the atrial segmentation was divided into 4 properties with distinct electrophysiological properties, electrotonic loading smoothes the differences in electrophysiological properties across the boundaries of these regions. This is exemplified in **Figure S7**, where APD_{90} measured in 3D tissue perpendicularly to the LA-PV border is shown to be much more smoothly varying than the single-cell APD_{90} values for the corresponding cell types. ERP values measured in 3D in the LA and PV are also shown to demonstrate how the changes in APD_{90} also affect ERP. Our previous study [18] has additionally shown that introducing smooth gradients in ionic conductances across such boundaries in 3D atrial model leads to minor changes in vulnerable windows compared to sharp borders (see supplementary Figure S14 in [18]).

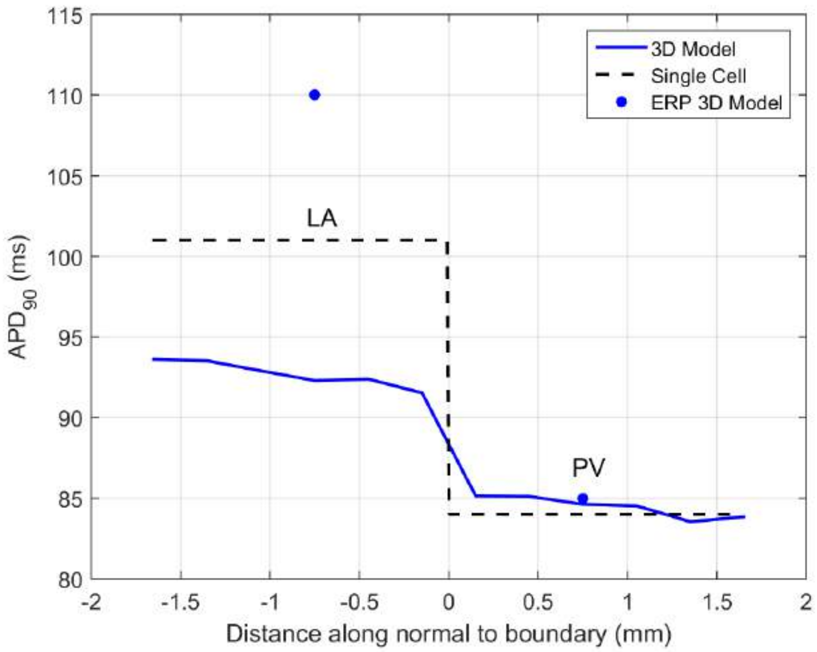

Figure S 7: Effect of electrotonic loading on APD_{90} heterogeneity. APD_{90} was measured across the LA-PV boundary in 3D (full blue line) and compared against single-cell APD_{90} values for corresponding cell types (dashed black line). ERPs (measured in 3D) for each of the tissue types are also shown (dark blue circles). Simulations were carried out at a BCL of 300 ms, with moderate ionic remodelling and CV reduction conditions.

2.2 Structural Remodelling Parameters

Anisotropic electrical conduction due to the fibre orientation field was incorporated into the model by setting different diffusion coefficients (eigenvalues of D) for the directions respectively parallel (D_{long}) and perpendicular (D_{trans}) to the fibre orientation at each voxel. Values for D_{long}, as well as for the anisotropy ratio $AR = (D_{long}/D_{trans})$ are stated in **Table S5**, for all atrial regions and for different degrees of structural remodelling associated with atrial fibrillation.

The values chosen for the diffusion anisotropy ratio (AR) correspond to anisotropy ratios in CV of 3.3 - 4.7. These lie between the very spread out experimental values of 1.3 ± 0.2 [19] and 5.2 ± 1.6 [20], 6.6 ± 2.4 [21] obtained in the right atrium or Bachmann's

bundle of healthy dogs at a physiological pacing rate.

$D_{long}(mm^2/ms); AR$	**RA**	**LA**	**BB-CT**	**PV**
**Baseline**	0.4; 10	0.4; 10	0.6; 16	0.4; 16
**CV Reduction**	0.2; 10	0.2; 10	0.4; 16	0.2; 16
**AR Increase**	0.2; 16	0.2; 22	0.4; 16	0.2; 22

Table S 5: Values for the longitudinal diffusion coefficient (D_{long}) and anisotropy ratio (AR) for each region of the atria. These are shown for three different stages of structural remodelling: baseline, CV reduction and additional AR increase.

Conduction velocity values measured in the 3D atrial model are shown in **Table S6** alongside corresponding experimental values measured in dogs, with which they show a good agreement. The activation time maps resulting from pacing in the sino-atrial node region at 350 ms are additionally shown in **Table S7**, alongside experimental activation times obtained in healthy dogs in similar conditions [22, 23]. Again, good agreement between the values provided by the model and experiments is observed.

$CV_{longitudinal}$ **(m/s)**	**Model**	**Canine Experiments**
**RA, no remodelling**	0.94	0.80-1.10 [24, 25, 26, 20]
**BB, no remodelling**	1.18	1.00-1.30 [25, 27, 28, 21]
**RA, after remodelling/RAP**	0.64	0.60-0.90 [25, 24, 29]

Table S 6: Longitudinal conduction velocity values ($CV_{longitudinal}$) measured in the 3D atrial model. Values from the current study computed at a BCL of 350 m are shown alongside corresponding experimental values measured experimentally in dogs.

**Region**	**Sakamoto et al, 2005 [22]**	**Hayashi et al, 1982 [23]**	**Model**
**SVC**	15 ms	10 ms	14 ms
**RAA**	25 ms	37 ms	38 ms
**IVC**	45 ms	35 ms	40 ms
**LBB**	35 ms	25 ms	28 ms
**LAA**	65 ms	55 ms	68 ms
**RSPV**	50 ms	40 ms	39 ms
**RIPV**	55 ms	30 ms	49 ms

Table S 7: Activation times in different regions when pacing from the sino-atrial node, at a BCL of 350 ms. SVC: superior vena cava; RAA: right atrial appendage; IVC: inferior vena cava; LBB: left portion of Bachmann's bundle; LAA: left atrial appendage; RSPV: right superior pulmonary vein; RIPV: right inferior pulmonary vein.

2.3 Computation of Vulnerable Windows

Vulnerable windows (VW) were computed in conditions that modelled different degrees of ionic and structural remodelling using an $S_1 - S_2$ protocol. VWs were defined as the range of S_2 values for which conduction block (for conduction block VW) or re-entry (for re-entry VW) occurred. VWs were scanned in steps of 1 ms. The outcomes of this analysis are shown in **Table 1**.

2.4 Intracellular Ca^{2+} in the 3D Model

Figure S8 shows the temporal evolution of the intracellular Ca^{2+} concentration (Cai) during fast pacing (BCL = 200 ms) from the left superior PV. Initial variation in Cai **Figure 8a** comes from the differences in g_{CaL} expression in different regions of the atria. (The single-cell model was paced 10 times at the same BCL to give adequate starting conditions for the 3D simulation.) In **Figure S8**, it can be seen that the CaT wave (left side of each panel) lags behind the depolarization wave (in red, on the right hand side of each panel) and that it has a very heterogeneous structure. We believe this is due to different electrotonic loadings in neighbouring voxels, which cause changes in each voxel's AP and plateau characteristics. These result in different CaT across each cell and give rise to the heterogeneous appearance of the Cai waves in **Figure S8**. These intra-regional differences are increased as additional depolarizing waves activate the atria (compare **Figure S8b** and **d**).

No intracellular Ca^{2+} overload was observed, with Cai showing similar levels to those in sinus rhythm conditions and never exceeding 380 nM. Moreover, even if intracellular Ca^{2+} overload was present, it would not necessarily have affected the dynamics of re-entry and AF, as shown in our previous simulations of 3D rabbit atrial tissue [30].

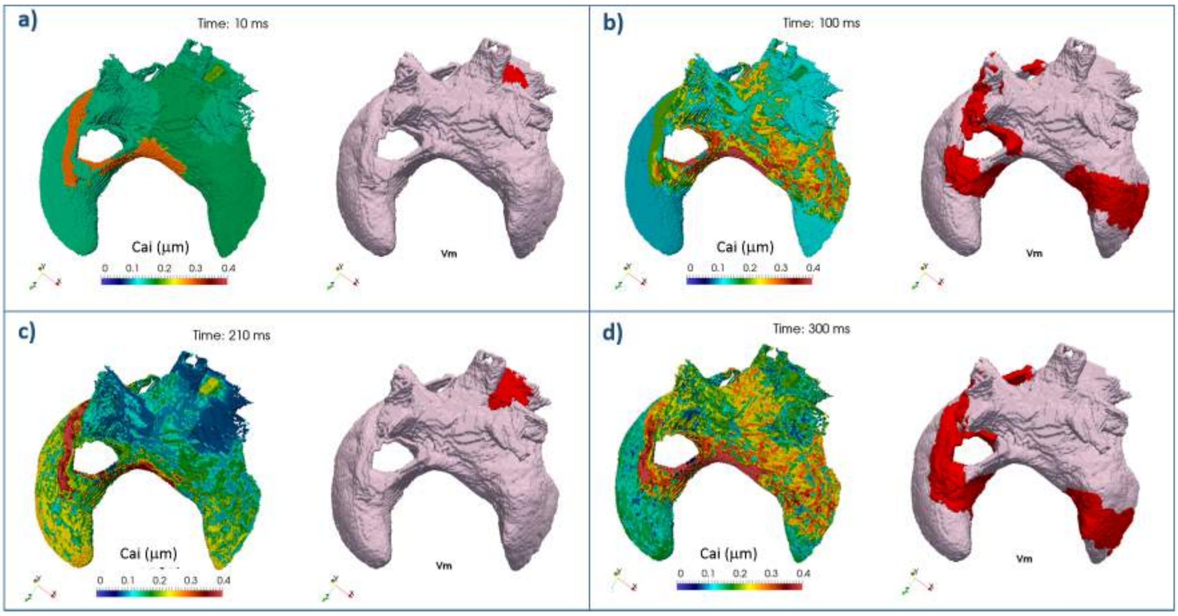

Figure S 8: Temporal evolution of the intracellular concentration of Ca^{2+} ($\boldsymbol{Cai}$) in the 3D model after fast pacing. Pacing was performed in the left superior PV, in conditions of CV reduction and moderate ionic remodelling. Impulses are applied at 0 and 200 ms. The depolarizing wavefront ($V > -20mV$) can be seen in red on the righthand side of each panel for the same time points as the Cai maps on the left hand side. Time since initial pacing: **a)** 10 ms, **b)** 100 ms, **c)** 210 ms, **d)** 300 ms.

2.5 Modelling Anti-Arrhythmic Drug Action

Markov models that incorporate state, time- and/or voltage-dependent blockade provide a very sophisticated model of anti-arrhythmic drug action. However, there are few atrial experimental data for multi-channel blockers, such as amiodarone and vernakalant, which would allow us to accurately parameterise these complex models of anti-arrhythmic drug action. For this reason, current modelling studies of multi-channel blockers rely on simple ionic channel conductance blockades (for example, [31, 32]). Given the lack of relevant experimental information, we believe the blockade model is the most sensible approximation for the current knowledge of their action.

The blockade factors used to model the effect of class-III drugs amiodarone and vernakalant at different concentrations are listed in **Table S8**, alongside IC_{50} values from the literature for each current affected by the drug.

Current	Amio 5 μM	Amio 10 μM	IC_{50} Amio (μM)	Verna 10 μM	Verna 30 μM	IC_{50} Verna (μM)
I_{Na}	0.90	0.81	4.8 [33], >3 [34]	1.00	0.90	107 ± 11 [35]
I_{CaL}	0.50	0.35	10.0 [36], 5.8 [37]	1	1	-
I_{Ks}	0.50	0.35	3.8 [32], 0.1 [34]	1	1	-
I_{Kr}	0.50	0.35	2.8 [38], 9.8 [39]	0.68	0.41	21 ± 3 [35]
I_{K1}	0.70	0.49	30 [34], >15 [40]	1	1	-
I_{to}	1	1	-	0.56	0.30	15 ± 2 [35]
I_{Kur}	1	1	-	0.55	0.30	13 ± 1 [35]
I_{KAch}	0.22	0.15	1.0 [34]	0.54	0.22	10 ± 1 [35]

Table S 8: Blockade factors for amiodarone and vernakalant. The values used to model 5 and 10 μM amiodarone (Amio) and 10 and 30 μM vernakalant (Verna) are accompanied by IC_{50} values from the literature for each current affected by the drug. Corresponding literature references are also shown.

The changes in action potential morphology in single-cell models following drug administration are depicted in **Figure S9a** for the RA model and different degrees of remodelling. **Figure S9b** and **c** show corresponding changes in APD_{90} following drug administration and corresponding differences in APD_{90} across different atrial regions. APD restitution curves for all cell types and stages of ionic remodelling can be seen in **Figure S10**.

The effect of anti-arrhythmic drugs on ERP and APD_{90} was compared against experimental results obtained in canine models. Our model accurately reproduces the relative changes in ERP and APD_{90} caused by drug administration, as can be seen in **Figure S11**.

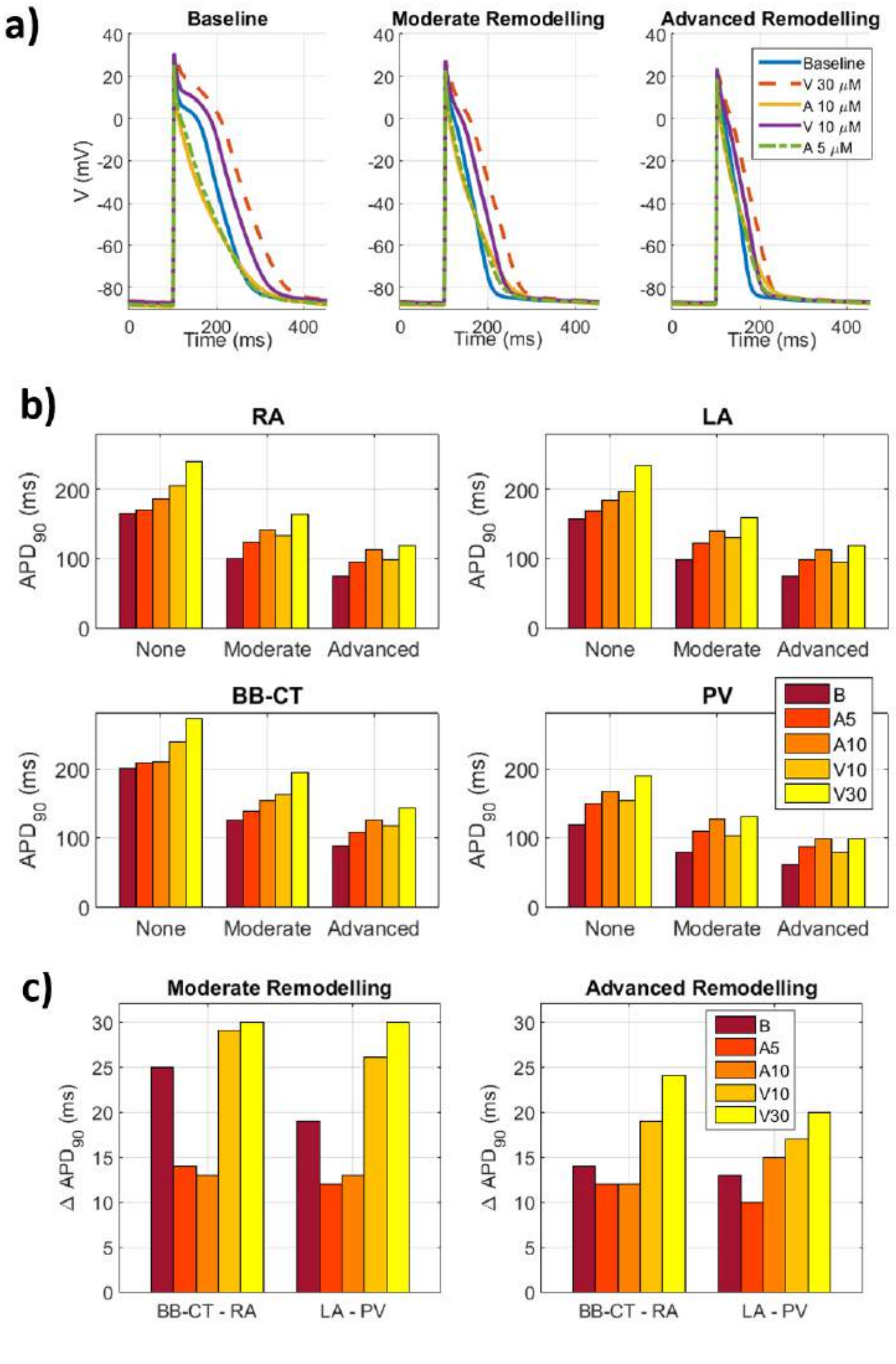

Figure S 9: Effect of anti-arrhythmic drugs on single cell action potentials. a) Action potentials for the right atrium, paced at 2 Hz, in the absence of drugs and after administration of vernakalant or amiodarone for different degrees of ionic remodelling. **b)** Absolute APD_{90} values in all cell types and **c)** differences in APD_{90} between right atrial tissues (BB-CT and RA) and left atrial tissues (PV and LA) for: different degrees of remodelling (none, moderate or advanced) at baseline (B) and after the application of either 5, 10 μM of amiodarone (A5, A10) or 10, 30 μM of vernakalant (V10, V30).

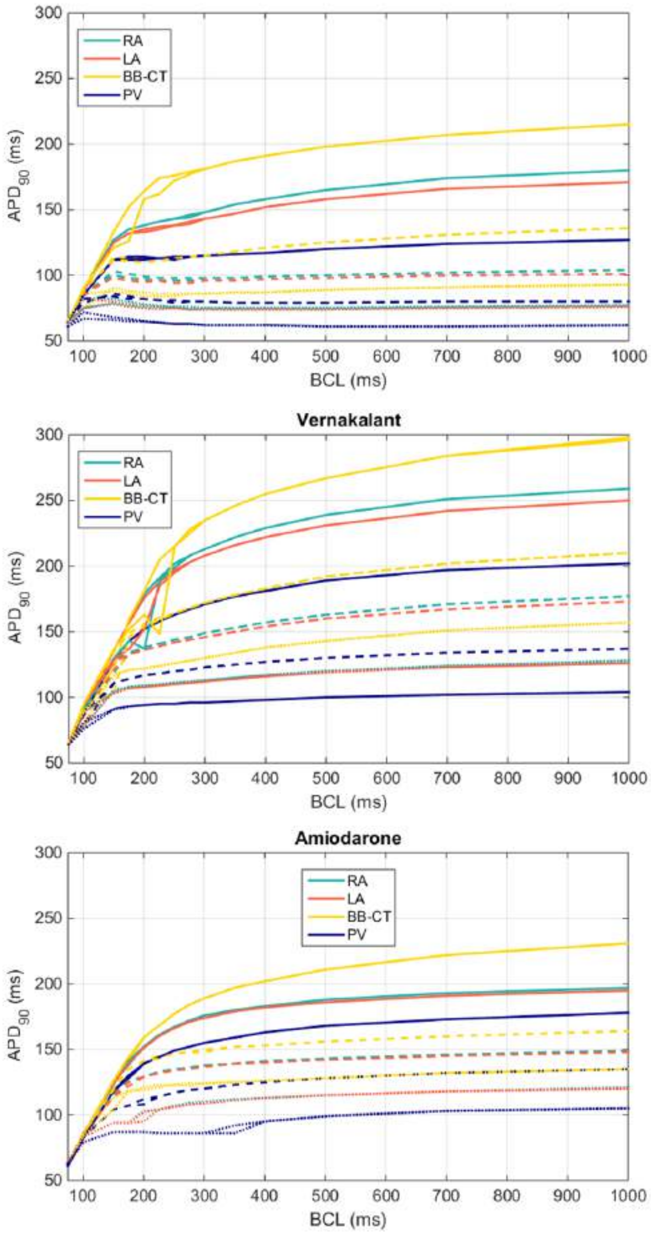

Figure S 10: APD_{90} **restitution curves.** APD_{90} restitution curves in baseline (full line), moderate (dashed line) and advanced (dotted line) ionic remodelling for all atrial cell types in baseline conditions (top panel) and after the administration of 30 μM of vernakalant (centre panel) or 10 μM amiodarone (bottom panel).

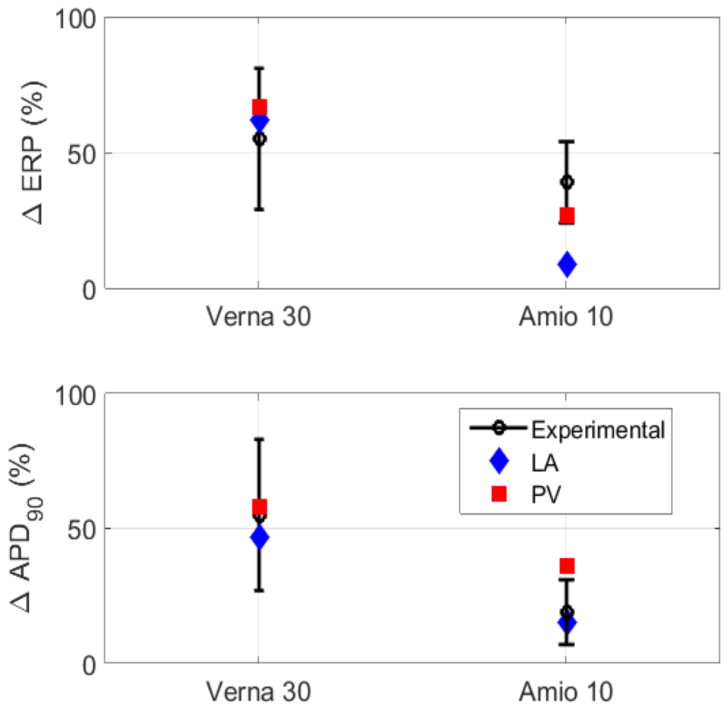

Figure S 11: Relative changes in ERP and APD_{90} induced by drug administration. Experimental data (black) is taken from [41] for the action of vernakalant 30 μM on healthy canine PVs and [42] for the action of chronic amiodarone on healthy canine RA cells. Simulations were carried out for the PV and LA cells using the described 1D cable model (for ERP calculations) and single-cell models (for APD_{90}) at a BCL of 500 ms, in the absence of any remodelling.

2.6 Conduction Velocity, APD_{90} and Effective Refractory Period Measurements

In the computed 3D APD_{90} atrial maps, diffusion coefficients matched those used in simulations of atrial fibrillation and pacing was performed in the sinoatrial node every 500 ms.

Conduction velocity restitution curves and effective refractory periods (ERPs) were computed in a cable with 100 elements (side: 0.02 mm, time step: 5 μs) using a 1D version of **Equation 1** with the same D_{long} as in the baseline 3D models. Both moderate and advanced remodelling conditions were considered. ERP was measured as the minimum pacing length for which conduction is still possible. Similar analyses were repeated under the action of 10μM of amiodarone and 30μM of vernakalant. It can be seen that ERP dispersion is small under the action of amiodarone in all conditions, and is much larger under the action of vernakalant.

3D maps of the magnitude of conduction velocity were also computed from activation time maps, as suggested in [43]. Briefly, activation time (AT) maps were computed by fast pacing in the LSPV at a BCL of 150 ms. The gradient of AT was computed and the

magnitude of CV, $||CV||$ calculated, on a voxel by voxel basis, as:

$$||CV|| = \frac{\Delta x}{||\nabla AT||} \tag{2}$$

where Δx denotes the intervoxel spacing (0.3 mm). Gradient calculations were performed in Paraview (Kitware, CA, USA). CV values computed using this method also include components perpendicular to the atrial surface and cannot therefore be directly compared to the epi- or endocardial CV measurements performed experimentally. They also depend on the pacing site, which was chosen to be in the PVs to best mimic AF conditions.These simulations were performed in moderate ionic remodelling and CV reduction conditions for: baseline (drug-free) models and also under the effect of amiodarone 10 and vernakalant 30.

2.7 Role of I_{K1} and I_{CaL} in Drug Action

We additionally investigated the contribution of two of the main ionic channels, I_{K1} and I_{CaL}, on drug action. To this end, we separately removed all I_{K1} and I_{CaL} block from the action of amiodarone 10 μM. We also added I_{K1} and I_{CaL} blocks (by a factor of 0.60) to the action of vernakalant 30 μM. These drug actions were studied in the same conditions as amiodarone and vernakalant, that is, after 5 s of activity, assuming instantaneous action, in CV reduction conditions and for two degrees of ionic remodelling. The outcome of these drug actions, 5 s after administration, is listed in **Table S9**.

**Ionic Remodel.**	**Baseline**	**Amio 10 - I_{K1} block**	**Amio 10 - I_{CaL} block**	**Verna 30 + I_{K1} block**	**Verna 30 + I_{CaL} block**
**Moderate**	PV: 2 rotors RA: wavelets	PV: 2 rotors RA: 1 rotor	All activity terminated	PV: 1 rotor RA: wavelets	PV: 1 rotor RA: wavelets
**Severe**	PV: 2 rotors LA: 1 rotor RA: 1 rotor, wavelets	PV: 2 rotors RA: 1 rotor, wavelets	All activity terminated	PV: 2 rotors RA: wavelets	PV: 2 rotors RA: 1 rotor

Table S 9: Outcome of 3D simulations after the administration of modified drug actions. Modified amiodarone 10 (Amio 10) and vernakalant 30 (Verna 30) drugs were applied are their outcome is compared to baseline (no drug) conditions. All simulations lasted 5 s. Blockade factors for additional I_{K1} and I_{CaL} blocks were 0.60.

We found that the action of either amiodarone or vernakalant could not be attributed to their inhibitory effect on a single ionic channel, such as I_{K1} [44]. To test our hypothesis that the most favourable drug actions were those leading to concomitant increases in ERP and decreases in ERP heterogeneity, we measured ERP in a 1D cable (as described above) in the same conditions as in **Table S9**. The respective full CV restitution curves are shown in **Figure S12**.

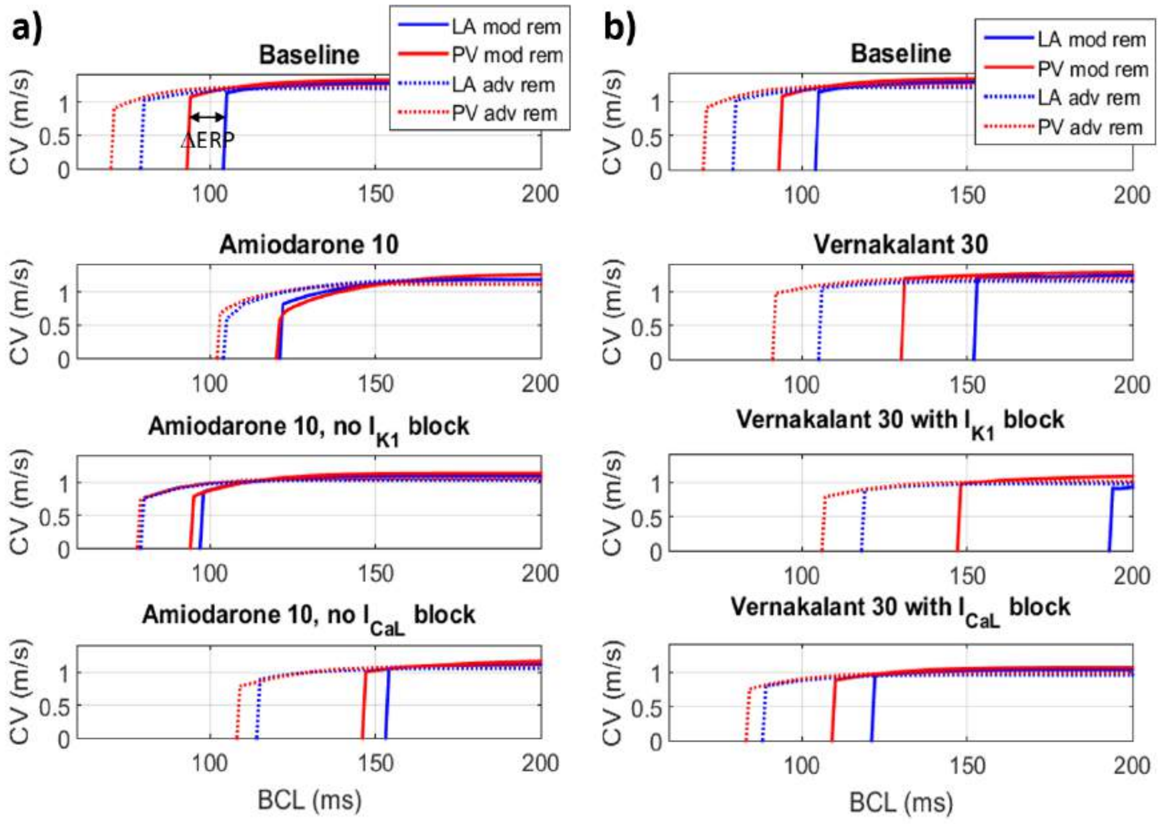

Figure S 12: Conduction velocity restitution curves. Conduction velocity restitution curves simulated in 1D for PV (red) and LA (blue) cells, in conditions of moderate (full lines) and severe remodelling (dashed lines). Panels show CV restitution curves for baseline values (top), in the presence of amiodarone 10 μM (**a), second panel**) and vernakalant 30 μM (**b), second panel**). The bottom two panels depict CV restitution curves for single-ionic channel modifications of amiodarone 10 μM (**a**)) or vernakalant 30 μM (**b**)).

In agreement with our hypothesis, we found that the most effective drug actions (amiodarone 10 μM, amiodarone 10 μM without I_{CaL} block) were linked to both an increase in ERP and a decrease in ERP dispersion. On the other hand, drug actions that increased ERP at the expense of ERP dispersion (all variants of vernakalant 30 μM) or decreased ERP at the expense of ERP values (amiodarone 10 μM without I_{K1} block) were less effective. This is summarised in **Table S10**.

The action of amiodarone in the absence of I_{K1} block, in which I_{CaL} block becomes dominant, can be used as a simple model of the action class-IV anti-arrhythmic drugs, such as verapamil. Verapamil has been found to reduce AF-induced remodelling [45], but performs poorly in the cardioversion of AF [46], in agreement with the findings in **Table S10**.

	Amiodarone 10	Amiodarone 10, no I_{K1} block	Amiodarone 10, no I_{CaL} block
ERP	increased	reduced	increased
Δ ERP	reduced	reduced	reduced
3D outcome	good	poor	good

	Vernakalant 30	Vernakalant 30, with I_{K1} block	Vernakalant 30, with I_{CaL} block
ERP	increased	increased	increased
Δ ERP	increased	increased	increased
3D outcome	poor	poor	poor

Table S 10: Relationship between the effective refractory period (ERP), its dispersion and the outcome of 3D simulations in the presence of the analysed drug actions. We show the changes in effective refractory period (ERP) and dispersion of ERP between the LA and PV cells effected by analysed drug actions (shown in **Figure S12**), as well as the qualitative outcome of 3D simulations (**Table S9**) for the same drug actions.

2.8 Atrial Activation Maps under Drug Action

Figure S13a-c shows the 3D atrial activation times over one period of AF at baseline and under the action of amiodarone and vernakalant. In baseline conditions, re-entrant activation around the LSPV took about 100 ms. Due to the effect of amiodarone on APD, the duration of the re-entrant activation increased to over 120 ms. This meant that the rotor's wavefront met refractory tissue near the PVs, resulting in its termination (dashed lines). Vernakalant prolonged the period of re-entry even more substantially to over 130 ms. This did not, however, terminate the re-entry due to slow conduction associated with this drug (see **Figure 6** in the manuscript). Moreover, the slow conduction across the CT seen with vernakalant (**Figure 6**) resulted in a conduction block (solid line) leading to the formation of two rotors in the RA. This arrhythmogenic conduction block did not occur with amiodarone, due to lower dispersion of APD and CV.

These activations were linked with the underlying fibre orientation **Figure S13d**, but not determined by it alone, as evidenced by the differences between amiodarone and vernakalant. We found the APD gradients to be more important in determining rotor dynamics, with the fibre orientation field modulating it.

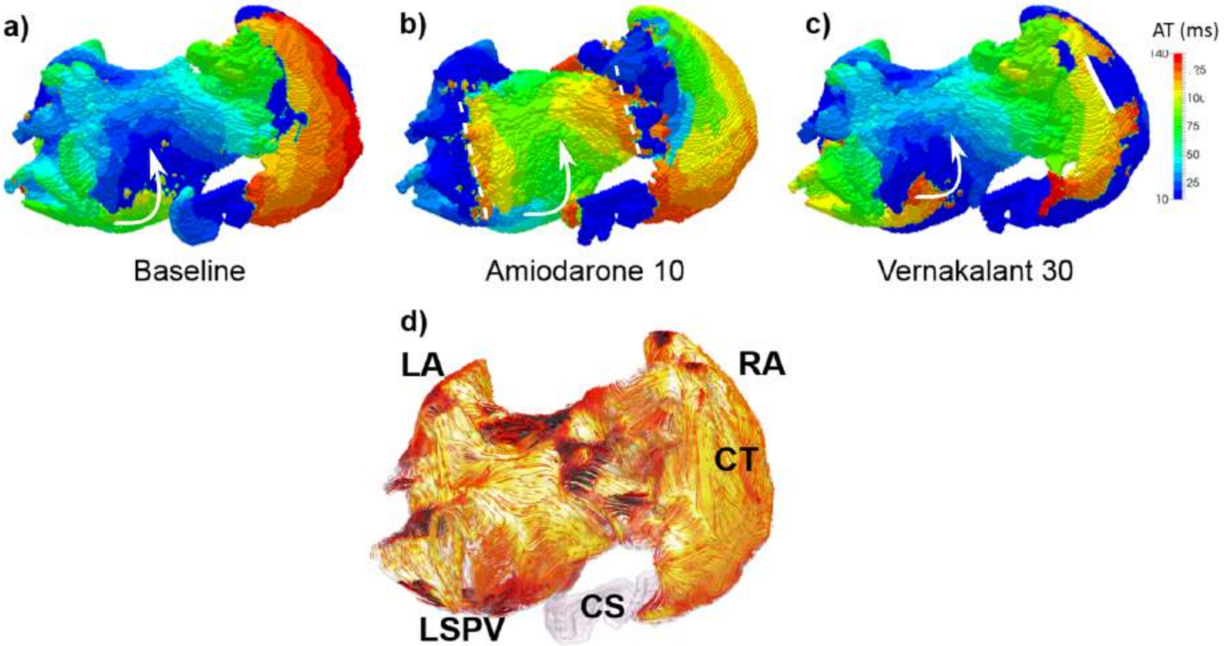

Figure S 13: Atrial activation times (ATs) in the presence of drugs in the 3D canine model. Superior-posterior view of the atria, showing ATs during AF (moderate ionic remodelling, CV reduction conditions) at **a)** baseline and after the application of **b)** amiodarone $10\mu M$ and **c)** vernakalant $30\mu M$. Panel **d)** shows the 3D fibre orientation in the same geometry. The rotor around the LSPV (arrow) is terminated near the PVs by amiodarone (dashed lines), whereas vernakalant creates an additional conduction block at the CT (full line).

3 Legends of Supplementary Videos

Legends explaining the conditions in which each supplementary video was created are listed below. In the cases where drug administration did not lead to termination of activity, the legends are accompanied by a screenshot of each video. In these, rotors and wavelets are marked by white arrows and labelled according to the region in which they reside. For all figures, the left side shows a posterior-superior view, whereas the right side depicts an anterior-inferior view of the atria.

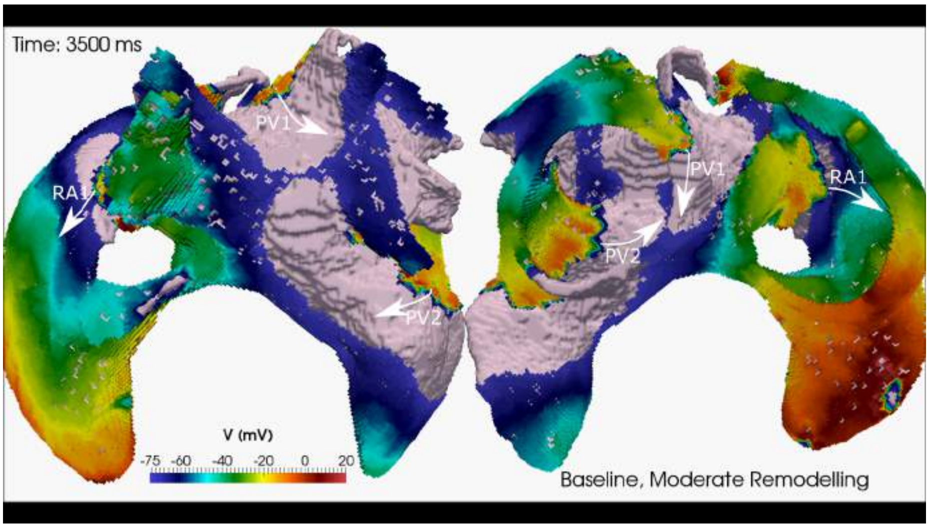

Video 1 - Spatiotemporal dynamics of electrical activity in the 3D atria in baseline (no drugs), CV reduction and moderate ionic remodelling conditions.

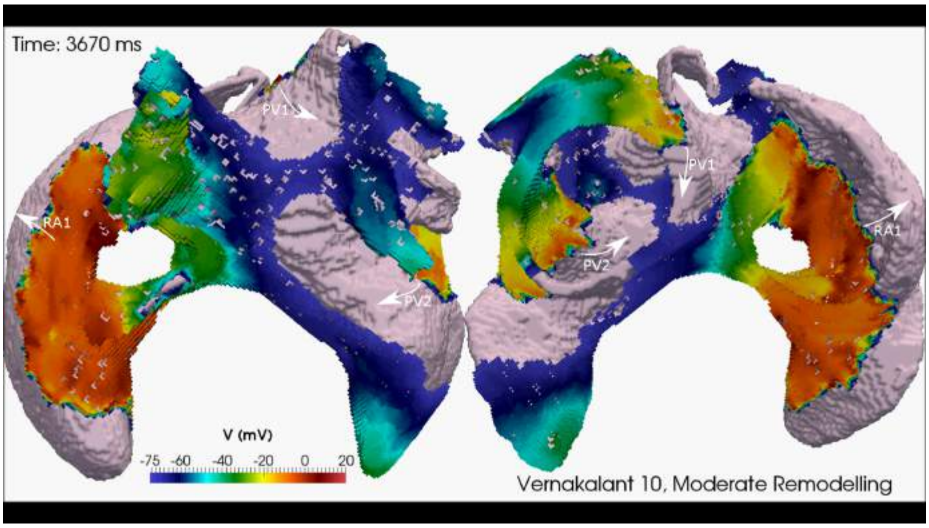

Video 2 - Spatiotemporal dynamics of electrical activity in the 3D atria after the administration of vernakalant (10 μM), in CV reduction and moderate ionic remodelling conditions.

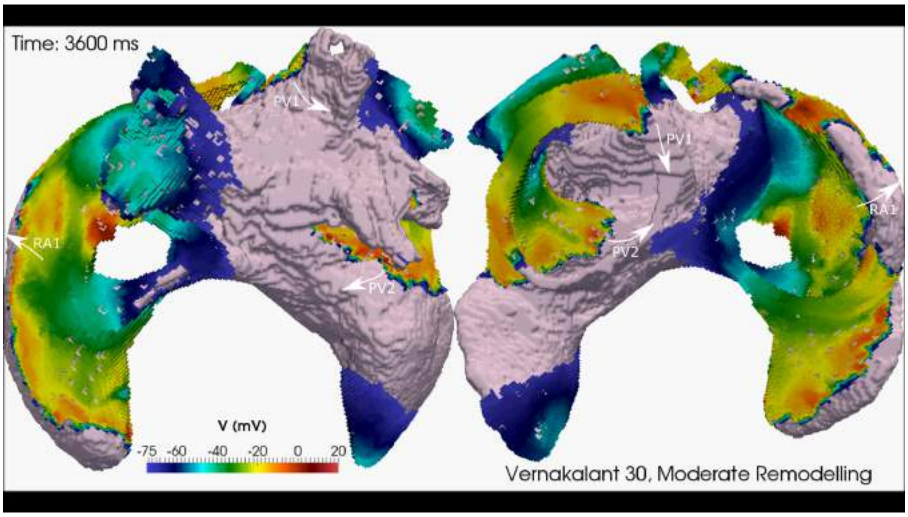

Video 3 - Spatiotemporal dynamics of electrical activity in the 3D atria after the administration of vernakalant (30 μM), in CV reduction and moderate ionic remodelling conditions.

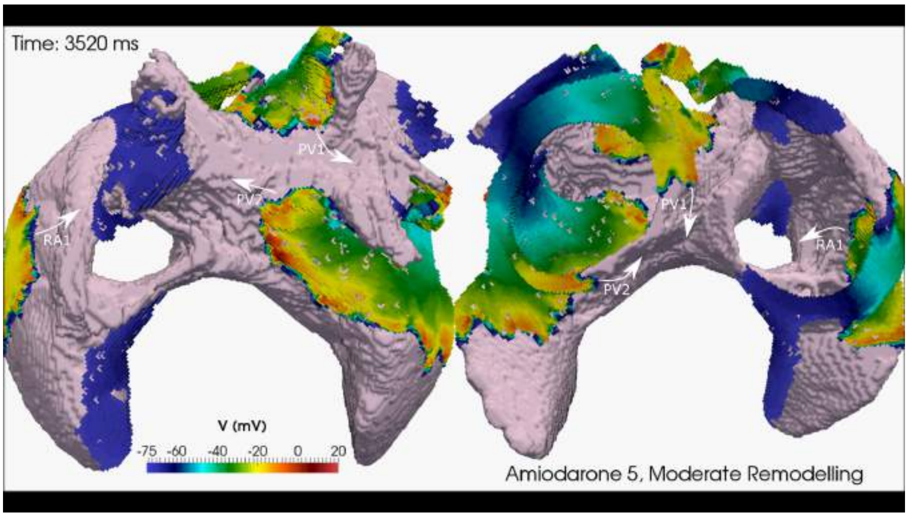

Video 4 - Spatiotemporal dynamics of electrical activity in the 3D atria after the administration of amiodarone (5 μM), in CV reduction and moderate ionic remodelling conditions.

Video 5 - Spatiotemporal dynamics of electrical activity in the 3D atria after the administration of amiodarone (10 μM), in CV reduction and moderate ionic remodelling conditions.

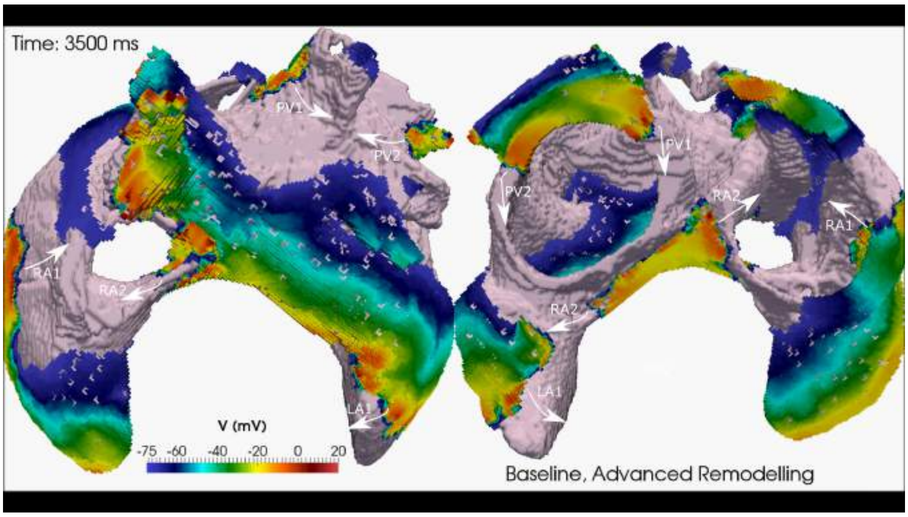

Video 6 - Spatiotemporal dynamics of electrical activity in the 3D atria in baseline (no drugs), CV reduction and advanced remodelling conditions.

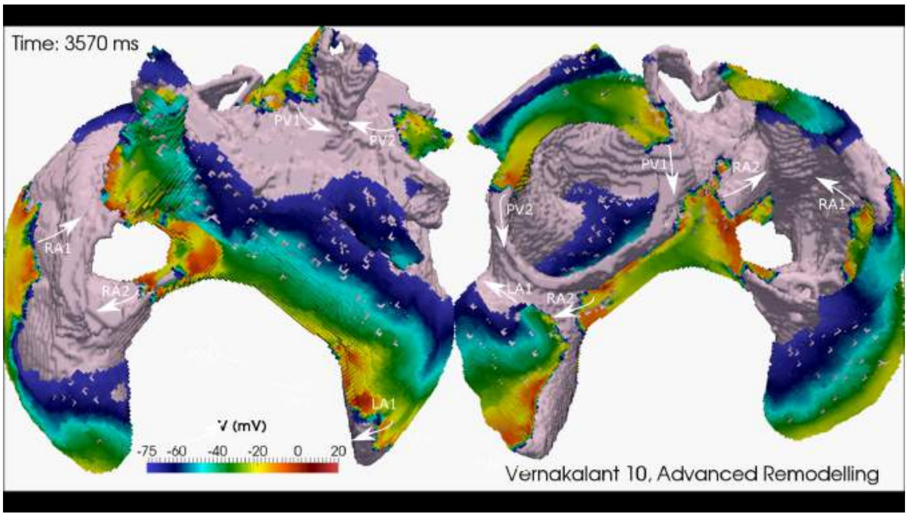

Video 7 - Spatiotemporal dynamics of electrical activity in the 3D atria after the administration of vernakalant (10 μM), in CV reduction and advanced remodelling conditions.

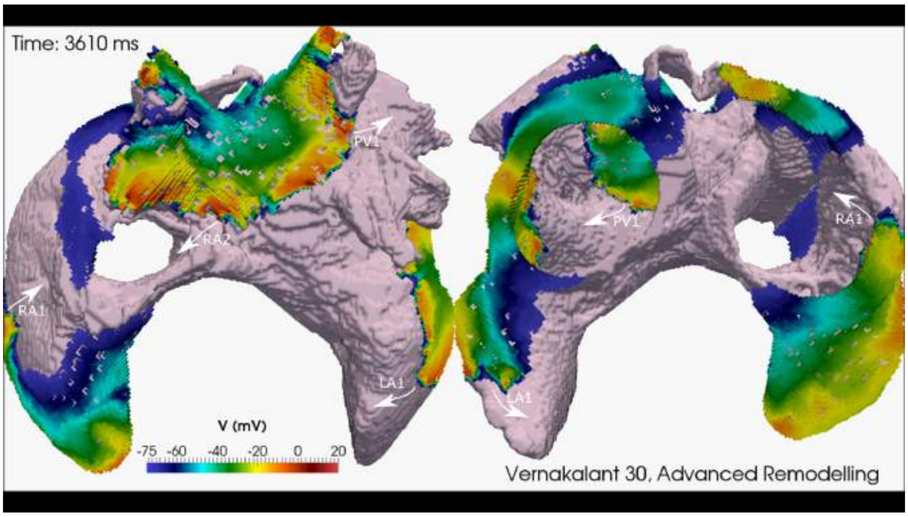

Video 8 - Spatiotemporal dynamics of electrical activity in the 3D atria after the administration of vernakalant (30 μM), in CV reduction and advanced remodelling conditions.

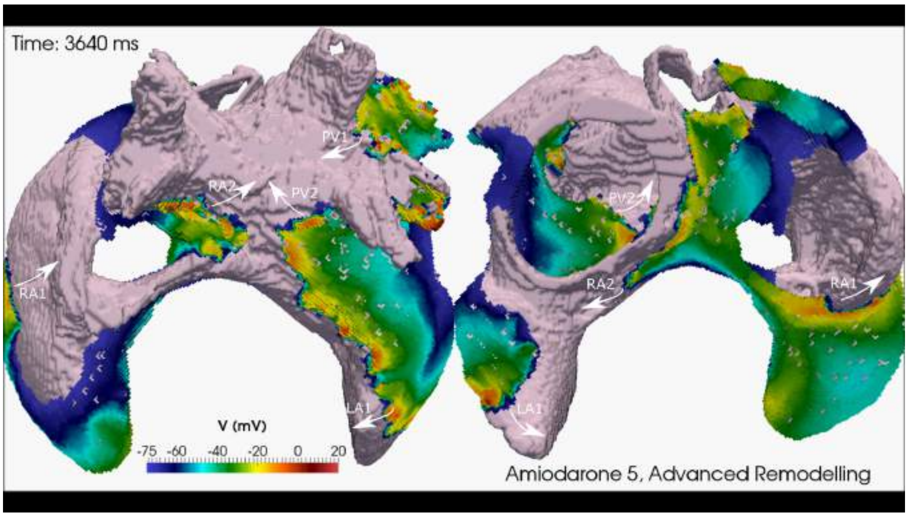

Video 9 - Spatiotemporal dynamics of electrical activity in the 3D atria after the administration of amiodarone (5 μM), in CV reduction and advanced remodelling conditions.

Video 10 - Spatiotemporal dynamics of electrical activity in the 3D atria after the administration of amiodarone (10 μM), in CV reduction and advanced remodelling conditions.

4 Mathematical Formulation of Ionic Currents

The total ionic current through the cell's membrane, I_{ion}, is given by the sum of all the ionic currents whose conductances are listed in **Table 1**:

$$I_{ion} = I_{Na}+I_{CaL}+I_{CaP}+I_{K1}+I_{to}+I_{Kur}+I_{bCl}+I_{Kr}+I_{Ks}+I_{bNa}+I_{bCa}+I_{NaCa}+I_{NaK}+I_{KAch} \tag{3}$$

For currents whose formulation is not listed below, the RNC model's formulation was used. All gating variables, y, obey the following ordinary differential equation:

$$\frac{dy}{dt} = \frac{y_\infty - y}{\tau_y} \tag{4}$$

4.1 I_{to}

$$I_{to} = g_{to} * o_a^3 * o_i * (V - E_k) \tag{5a}$$

$$o_{a\infty} = \left(1 + exp\left(\frac{V - 12}{-11.5}\right)\right)^{-\frac{1}{3}} \tag{5b}$$

$$\tau_{oa} = \frac{0.4}{exp\left(\frac{V-15}{20}\right)} \tag{5c}$$

$$o_{i\infty} = \left(1 + exp\left(\frac{V + 31}{6.45}\right)\right)^{-1} \tag{5d}$$

$$\tau_{oi} = \left(\left(1.2 + exp\left(\frac{V + 95.2}{5.85}\right)\right)^{-1} + \left(9.54 + exp\left(\frac{V - 48}{-20}\right)\right)^{-1}\right)^{-1} \tag{5e}$$

4.2 I_{Kur}

$$I_{Kur} = g_{Kur} * f_{Kur} * u_a^3 * u_i * (V - E_k) \tag{6a}$$

$$f_{Kur} = 1 + \frac{3}{1 + exp\left(-\frac{V-14}{6}\right)} \tag{6b}$$

$$u_{a\infty} = \left(1 + exp\left(-\frac{V + 2.81}{9.49}\right)\right)^{-\frac{1}{3}} \tag{6c}$$

$$\tau_{ua} = \left(\frac{1.47}{exp\left(\frac{V+33.2}{-30.63}\right) + exp\left(\frac{V-27.6}{-30.65}\right)} + \frac{0.42}{exp\left(\frac{V+26.64}{2.49}\right) + exp\left(\frac{V+44.41}{20.36}\right)}\right)^{-1} \tag{6d}$$

$$u_{i\infty} = \left(1 + exp\left(\frac{V - 99.45}{27.48}\right)\right)^{-1} \tag{6e}$$

$$\tau_{ui} = \left(\left(21 + exp\left(-\frac{V - 185}{28}\right)\right)^{-1} + exp\left(\frac{V - 158}{16}\right)\right)^{-1} \tag{6f}$$

4.3 I_{Kr}

$$I_{Kr} = g_{kr} * f_{kr} * x_r * (V - E_k) \tag{7a}$$

$$x_{r\infty} = \left(1 + exp\left(\frac{V + 7.654}{-5.377}\right)\right)^{-1} \tag{7b}$$

$$\tau_{xr} = \left(\frac{0.04 * (V - 248)}{1 - exp\left(-\frac{V-248}{28}\right)} + \frac{0.028 * (V + 163)}{exp\left(\frac{V+163}{21}\right) - 1}\right)^{-1} \tag{7c}$$

$$\textbf{RA, BB-CT: } f_{kr} = 0.6 + \left(0.5 + exp\left(\frac{V - 26}{20.4}\right)\right)^{-1} \tag{7d}$$

$$\textbf{LA, PV: } f_{kr} = 0.6 + \left(0.5 + 0.5 * exp\left(\frac{V + 8}{24.4}\right)\right)^{-1} \tag{7e}$$

4.4 I_{Ks}

$$I_{Ks} = g_{ks} * x_s^2 * (V - E_k) \tag{8a}$$

$$x_{s\infty} = \left(1 + exp\left(-\frac{V - 13}{12}\right)\right)^{-1/2} \tag{8b}$$

$$\tau_{xs} = \left(\frac{1 \times 10^{-5} * (V + 28.5)}{1 - exp\left(-\frac{V+28.5}{115}\right)} + \frac{2.3 \times 10^{-4} * (V + 28.5)}{exp\left(\frac{V+28.5}{3.3}\right) - 1}\right)^{-1} \tag{8c}$$

4.5 I_{K1}

LA, RA, BB-CT: $$I_{K1} = g_{K1} * \frac{V - E_k}{1 + exp\,(0.072 * (V - E_k))} \tag{9a}$$

PV: $$I_{K1} = g_{K1} * \frac{V - E_k}{0.66 + exp\,(0.078 * (V - E_k - 18))} \tag{9b}$$

4.6 I_{CaL}

$$I_{CaL} = g_{CaL} * d * f * f_{ca} * (V - 60) \tag{10a}$$

$$d_\infty = \left(1 + exp\left(-\frac{V + 2}{5}\right)\right)^{-1} \tag{10b}$$

$$\tau_d = \frac{1 - exp\left(-\frac{V+10}{6.24}\right)}{0.035 * (V + 10) * \left(1 + exp\left(-\frac{V+10}{6.24}\right)\right)} \tag{10c}$$

$$f_\infty = \left(1 + exp\left(\frac{V + 34}{6.3}\right)\right)^{-1} \tag{10d}$$

$$\tau_f = \frac{400}{1 + 4.5 * exp\left(-7 \times 10^{-4} * (V - 9)^2\right)} \tag{10e}$$

$$f_{ca\infty} = 0.29 + \frac{0.8}{1 + exp\left(\frac{[Ca]_i - 1.2 \times 10^{-4}}{6 \times 10^{-5}}\right)} \tag{10f}$$

$$\tau_{fca} = 2 \tag{10g}$$

4.7 I_{KAch}

$$I_{KAch} = g_{KAch} * x_a * f_{KAch} * (V - E_k) \tag{11a}$$

$$f_{KAch} = \left(0.1 + exp\left(7.8 \times 10^{-2}\,(V + 19.97)\right)\right)^{-1} \tag{11b}$$

$$x_{a\infty} = \left(1 + exp\left(\frac{V + 93}{15.2}\right)\right)^{-1} \tag{11c}$$

$$\tau_{xa} = 360 + 130\left(1 - exp\left(-\frac{V + 130}{50}\right)\right) \tag{11d}$$

References

[1] Ehrlich, J.R., Cha, T.J., Zhang, L., Chartier, D., Melnyk, P., Hohnloser, S.H., Nattel, S.: Cellular electrophysiology of canine pulmonary vein cardiomyocytes: action potential and ionic current properties. J. Physiol. **551** (sep 2003) 801–13

[2] Li, D.K.B., Zhang, L., Kneller, J., Nattel, S.: Potential Ionic Mechanism for Repolarization Differences Between Canine Right and Left Atrium. Circ. Res. **88**(11) (may 2001) 1168–1175

[3] Feng, J., Yue, L., Wang, Z., Nattel, S.: Ionic Mechanisms of Regional Action Potential Heterogeneity in the Canine Right Atrium. Circ. Res. **83**(5) (sep 1998) 541–551

[4] De La Fuente, M.G., Barana, A., Gómez, R., Amorós, I., Dolz-Gaitón, P., Sacristán, S., Atienza, F., Pita, A., Pinto, A., Fernández-Avilés, F., Caballero, R., Tamargo, J., Delpón, E.: Chronic atrial fibrillation up-regulates Beta1-Adrenoceptors affecting repolarizing currents and action potential duration. Cardiovasc. Res. **97**(2) (2013) 379–388

[5] Voigt, N., Trausch, A., Knaut, M., Matschke, K., Varró, a., Van Wagoner, D.R., Nattel, S., Ravens, U., Dobrev, D.: Left-to-right atrial inward rectifier potassium current gradients in patients with paroxysmal versus chronic atrial fibrillation. Circ. Arrhythm. Electrophysiol. **3**(5) (oct 2010) 472–80

[6] Ehrlich, J.R., Cha, T.J., Zhang, L., Chartier, D., Villeneuve, L., Hébert, T.E., Nattel, S.: Characterization of a hyperpolarization-activated time-dependent potassium current in canine cardiomyocytes from pulmonary vein myocardial sleeves and left atrium. J. Physiol. **557**(Pt 2) (jun 2004) 583–97

[7] Cha, T.J., Ehrlich, J.R., Zhang, L., Chartier, D., Leung, T.K., Nattel, S.: Atrial tachycardia remodeling of pulmonary vein cardiomyocytes: comparison with left atrium and potential relation to arrhythmogenesis. Circulation **111**(6) (feb 2005) 728–35

[8] Voigt, N., Maguy, A., Yeh, Y., Qi, X.Y., Ravens, U., Dobrev, D., Nattel, S.: Changes in I K, ACh single-channel activity with atrial tachycardia remodelling in canine atrial cardiomyocytes. Cardiovasc. Res. **77**(1) (jan 2008) 35–43

[9] Wakili, R., Yeh, Y., Yan Qi, X., Greiser, M., Chartier, D., Nishida, K., Maguy, A., Villeneuve, L., Boknik, P., Voigt, N., Krysiak, J., Kääb, S., Ravens, U., Linke, W.a., Stienen, G.J.M., Shi, Y., Tardif, J.C., Schotten, U., Dobrev, D., Nattel, S.: Multiple potential molecular contributors to atrial hypocontractility caused by atrial tachycardia remodeling in dogs. Circ. Arrhythm. Electrophysiol. **3**(5) (oct 2010) 530–41

[10] Coutu, P., Chartier, D., Nattel, S.: Comparison of Ca2+-handling properties of canine pulmonary vein and left atrial cardiomyocytes. Am. J. Physiol. Heart Circ. Physiol. **291**(5) (nov 2006) H2290–300

[11] Greiser, M., Kerfant, B.G., Williams, G.S.B., Voigt, N., Harks, E., Dibb, K.M., Giese, A., Meszaros, J., Verheule, S., Ravens, U., Allessie, M.A., Gammie, J.S., van der Velden, J., Lederer, W.J., Dobrev, D., Schotten, U.: Tachycardia-induced silencing of subcellular Ca2+ signaling in atrial myocytes. J. Clin. Invest. **124**(11) (nov 2014) 4759–72

[12] Ramirez, R., Nattel, S., Courtemanche, M.: Mathematical analysis of canine atrial action potentials : rate , regional factors , and electrical remodeling. Am J Physiol Hear. Circ Physiol **279**(4) (oct 2000) 1522–1539

[13] Yue, L., Feng, J., Gaspo, R., Li, G.R., Wang, Z., Nattel, S.: Ionic Remodeling Underlying Action Potential Changes in a Canine Model of Atrial Fibrillation. Circ. Res. **81**(4) (oct 1997) 512–525

[14] Burashnikov, A., Mannava, S., Antzelevitch, C.: Transmembrane action potential heterogeneity in the canine isolated arterially perfused right atrium: effect of IKr and IKur/Ito block. Am. J. Physiol. Heart Circ. Physiol. **286**(6) (jun 2004) H2393–400

[15] Chen, Y.J., Chen, Y.C., Tai, C.T., Yeh, H.I., Lin, C.I., Chen, S.A.: Angiotensin II and angiotensin II receptor blocker modulate the arrhythmogenic activity of pulmonary veins. Br. J. Pharmacol. **147**(1) (jan 2006) 12–22

[16] Gaspo, R., Bosch, R.F., Bou-Abboud, E., Nattel, S.: Tachycardia-Induced Changes in Na+ Current in a Chronic Dog Model of Atrial Fibrillation. Circ. Res. **81**(6) (dec 1997) 1045–1052

[17] Cha, T.J., Ehrlich, J.R., Zhang, L., Nattel, S.: Atrial ionic remodeling induced by atrial tachycardia in the presence of congestive heart failure. Circulation **110**(12) (sep 2004) 1520–6

[18] Colman, M., Aslanidi, O., Kharche, S., Boyett, M., Garratt, C.J., Hancox, J.C., Zhang, H.: Pro-arrhythmogenic effects of atrial fibrillation-induced electrical remodelling: insights from the three-dimensional virtual human atria. J. Physiol. **591**(Pt 17) (sep 2013) 4249–72

[19] Wu, T.J., Yashima, M., Xie, F., Athill, C.A., Kim, Y.H., Fishbein, M., Qu, Z., Garfinkel, A., Weiss, J.N., Karagueuzian, H.S., Chen, P.S.: Role of Pectinate Muscle Bundles in the Generation and Maintenance of Intra-atrial Reentry : Potential Implications for the Mechanism of Conversion Between Atrial Fibrillation and Atrial Flutter. Circ. Res. **83**(4) (aug 1998) 448–462

[20] Koura, T.: Anisotropic Conduction Properties in Canine Atria Analyzed by High-Resolution Optical Mapping: Preferential Direction of Conduction Block Changes From Longitudinal to Transverse With Increasing Age. Circulation **105**(17) (apr 2002) 2092–2098

[21] Dolber, P.C., Spach, M.S.: Structure of canine Bachmann's bundle related to propagation of excitation. Am J Physiol Hear. Circ Physiol **257**(5) (nov 1989) H1446–1457

[22] Sakamoto, S.I., Nitta, T., Ishii, Y., Miyagi, Y., Ohmori, H., Shimizu, K.: Interatrial electrical connections: the precise location and preferential conduction. J. Cardiovasc. Electrophysiol. **16**(10) (oct 2005) 1077–86

[23] Hayashi, H., Lux, R.L., Wyatt, R.F., Burgess, M.J., Abildskov, J.A.: Relation of canine atrial activation sequence to anatomic landmarks. Am. J. Physiol. **242**(3) (mar 1982) H421–H428

[24] Gaspo, R., Bosch, R.F., Talajic, M., Nattel, S.: Functional Mechanisms Underlying Tachycardia-Induced Sustained Atrial Fibrillation in a Chronic Dog Model. Circulation **96**(11) (dec 1997) 4027–4035

[25] Li, D.K.B., Fareh, S., Leung, T.K., Nattel, S.: Promotion of Atrial Fibrillation by Heart Failure in Dogs : Atrial Remodeling of a Different Sort. Circulation **100**(1) (jul 1999) 87–95

[26] Guerra, J.M.: Effects of the Gap Junction Modifier Rotigaptide (ZP123) on Atrial Conduction and Vulnerability to Atrial Fibrillation. Circulation **114**(2) (jul 2006) 110–118

[27] Spach, M.S., Miller, W.T., Dolber, P.C., Kootsey, J.M., Sommer, J.R., Mosher, C.E.: The functional role of structural complexities in the propagation of depolarization in the atrium of the dog. Cardiac conduction disturbances due to discontinuities of effective axial resistivity. Circ. Res. **50**(2) (feb 1982) 175–191

[28] Rensma, P.L., Allessie, M.A., Lammers, W.J., Bonke, F.I., Schalij, M.: Length of excitation wave and susceptibility to reentrant atrial arrhythmias in normal conscious dogs. Circ. Res. **62**(2) (feb 1988) 395–410

[29] Kumagai, K., Nakashima, H., Urata, H., Gondo, N., Arakawa, K., Saku, K.: Effects of angiotensin II type 1 receptor antagonist on electrical and structural remodeling in atrial fibrillation. J. Am. Coll. Cardiol. **41**(12) (jun 2003) 2197–2204

[30] Aslanidi, O., Boyett, M., Dobrzynski, H., Li, J., Zhang, H.: Mechanisms of transition from normal to reentrant electrical activity in a model of rabbit atrial tissue: interaction of tissue heterogeneity and anisotropy. Biophys. J. **96**(3) (feb 2009) 798–817

[31] Mirams, G.R., Cui, Y., Sher, A., Fink, M., Cooper, J., Heath, B.M., McMahon, N.C., Gavaghan, D.J., Noble, D.: Simulation of multiple ion channel block provides improved early prediction of compounds' clinical torsadogenic risk. Cardiovasc. Res. **91**(1) (jul 2011) 53–61

[32] Loewe, A., Lutz, Y., Wilhelms, M., Sinnecker, D., Barthel, P., Scholz, E., Doessel, O., Schmidt, G., Seemann, G.: In-silico assessment of the dynamic effects of amiodarone and dronedarone on human atrial patho-electrophysiology. Europace **16 Suppl 4**(suppl_4) (nov 2014) iv30–iv38

[33] Lalevée, N., Barrère-lemaire, S., Gautier, P., Nargeot, J., Richard, S.: Effects of Amiodarone and Dronedarone on Voltage-Dependent Sodium Current in Human Cardiomyocytes. J. Cardiovasc. Electrophysiol. **14**(8) (aug 2003) 885–890

[34] Patel, C., Yan, G.X., Kowey, P.R.: Dronedarone. Circulation **120**(7) (aug 2009) 636–44

[35] Fedida, D.: Vernakalant (RSD1235): a novel, atrial-selective antifibrillatory agent. Expert Opin. Investig. Drugs **16**(4) (apr 2007) 519–532

[36] Hancox, J.C.: Amiodarone blocks L-type calcium current in single myocytes isolated from the rabbit atrioventricular node. Gen. Pharmacol. Vasc. Syst. **29**(3) (sep 1997) 429–435

[37] Nishimura, M., Follmer, C., Singer, D.: Amiodarone blocks calcium current in single guinea pig ventricular myocytes. J. Pharmacol. Exp. Ther. **251**(2) (nov 1989) 650–659

[38] Kamiya, K., Nishiyama, A., Yasui, K., Hojo, M., Sanguinetti, M.C., Kodama, I.: Short- and Long-Term Effects of Amiodarone on the Two Components of Cardiac Delayed Rectifier K+ Current. Circulation **103**(9) (mar 2001) 1317–1324

[39] Kiehn, J., Thomas, D.L., Karle, C.A., Schöls, W., Kübler, W.: Inhibitory effects of the class III antiarrhythmic drug amiodarone on cloned HERG potassium channels. Naunyn. Schmiedebergs. Arch. Pharmacol. **359**(3) (mar 1999) 212–9

[40] Sato, R., Koumi, S., Singer, D., Hisatome, I., Jia, H., Eager, S., Wasserstrom, J.: Amiodarone blocks the inward rectifier potassium channel in isolated guinea pig ventricular cells. J. Pharmacol. Exp. Ther. **269**(3) (jun 1994) 1213–1219

[41] Sicouri, S., Pourrier, M., Gibson, J.K., Lynch, J.J., Antzelevitch, C.: Comparison of electrophysiological and antiarrhythmic effects of vernakalant, ranolazine, and sotalol in canine pulmonary vein sleeve preparations. Heart Rhythm **9**(3) (mar 2012) 422–9

[42] Sicouri, S., Carlsonn, L., Antzelevitch, C.: Electrophysiologic and antiarrhythmic effects of AZD1305 in canine pulmonary vein sleeves. J. Pharmacol. Exp. Ther. **334**(1) (jul 2010) 255–9

[43] Salama, G., Kanai, A., Efimov, I.R.: Subthreshold stimulation of Purkinje fibers interrupts ventricular tachycardia in intact hearts. Experimental study with voltage-sensitive dyes and imaging techniques. Circ. Res. **74**(4) (apr 1994) 604–619

[44] Filgueiras-Rama, D., Martins, R.P., Mironov, S., Yamazaki, M., Calvo, C.J., Ennis, S.R., Bandaru, K., Noujaim, S.F., Kalifa, J., Berenfeld, O., Jalife, J.: Chloroquine terminates stretch-induced atrial fibrillation more effectively than flecainide in the sheep heart. Circ. Arrhythmia Electrophysiol. **5**(3) (jun 2012) 561–570

[45] Tieleman, R.G., De Langen, C., Van Gelder, I.C., de Kam, P.J., Grandjean, J., Bel, K.J., Wijffels, M.C., Allessie, M.A., Crijns, H.J.: Verapamil reduces tachycardia-induced electrical remodeling of the atria. Circulation **95**(7) (apr 1997) 1945–53

[46] Suttorp, M.J., Kingma, J.H., Lie-A-Huen, L., Mast, E.G.: Intravenous flecainide versus verapamil for acute conversion of paroxysmal atrial fibrillation or flutter to sinus rhythm. Am. J. Cardiol. **63**(11) (mar 1989) 693–6
