# Supplementary figures and images for: Atrial Heterogeneity Generates Re-entrant Substrate during Atrial Fibrillation and Anti-arrhythmic Drug Action: Mechanistic Insights from Canine Atrial Models
Source: PLoS Comput Biol. 2016 Dec 16;12(12):e1005245. doi: 10.1371/journal.pcbi.1005245 (PMC5161306; doi:10.1371/journal.pcbi.1005245)

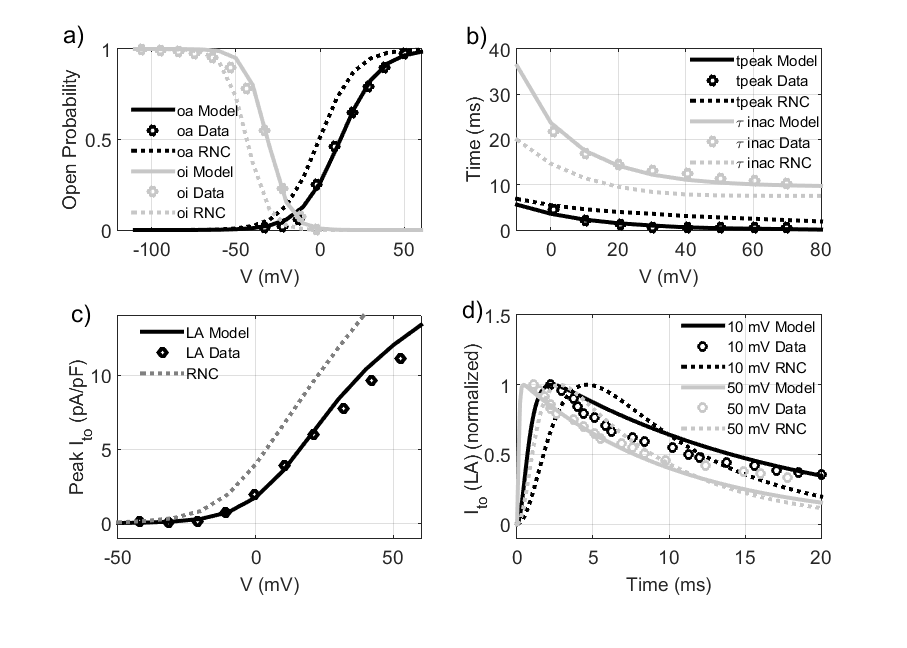

Supplement: S1 Fig — a) Steady-state values of the activation (oa) and inactivation (oi) variables as a function of membrane potential. b) Time to peak (tpeak) and inactivation time constant (τinac) as a function of membrane potential. c) Peak current-voltage relationship. d) Normalised current as a function of time for voltage steps of +50 and +10 mV (from -50 mV) as a function of time. (Experimental data for the LA cell taken from Ehrlich et al., 2003; Li et al., 2001.) (PNG) [file pcbi.1005245.s002.png]

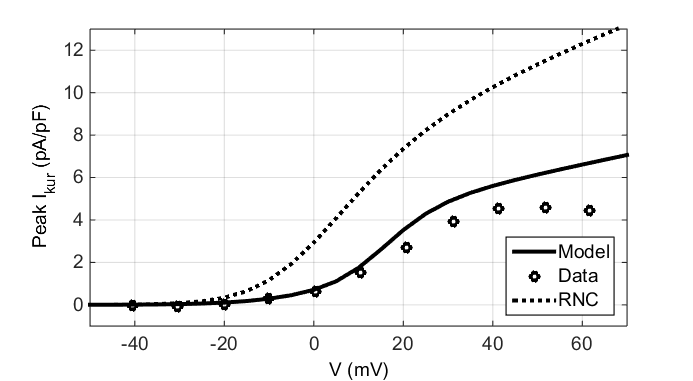

Supplement: S2 Fig — Peak current-voltage relationship. (Experimental data (for the LA and RA cells) is taken from Li et al., 2001.) (PNG) [file pcbi.1005245.s003.png]

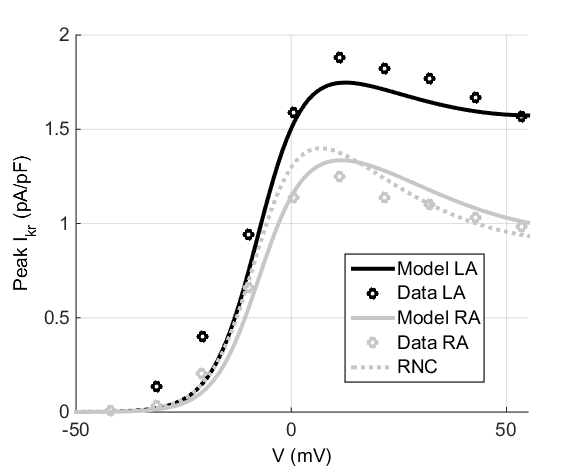

Supplement: S3 Fig — Peak current-voltage relationship. (Experimental data (for the LA and RA cells) is taken from Li et al., 2001.) (PNG) [file pcbi.1005245.s004.png]

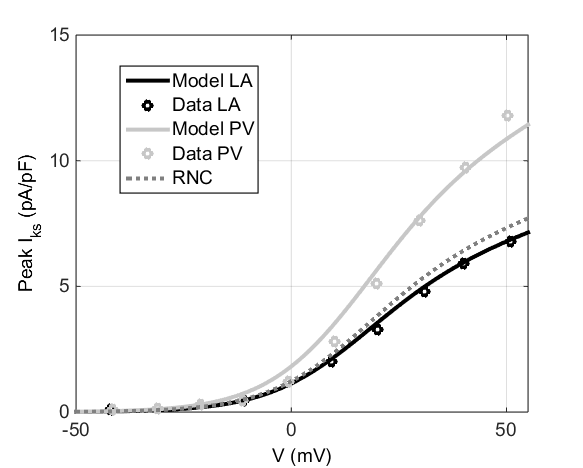

Supplement: S4 Fig — Peak current-voltage relationship. (Experimental data for the LA and RA cells is taken from Ehrlich et al., 2003.) (PNG) [file pcbi.1005245.s005.png]

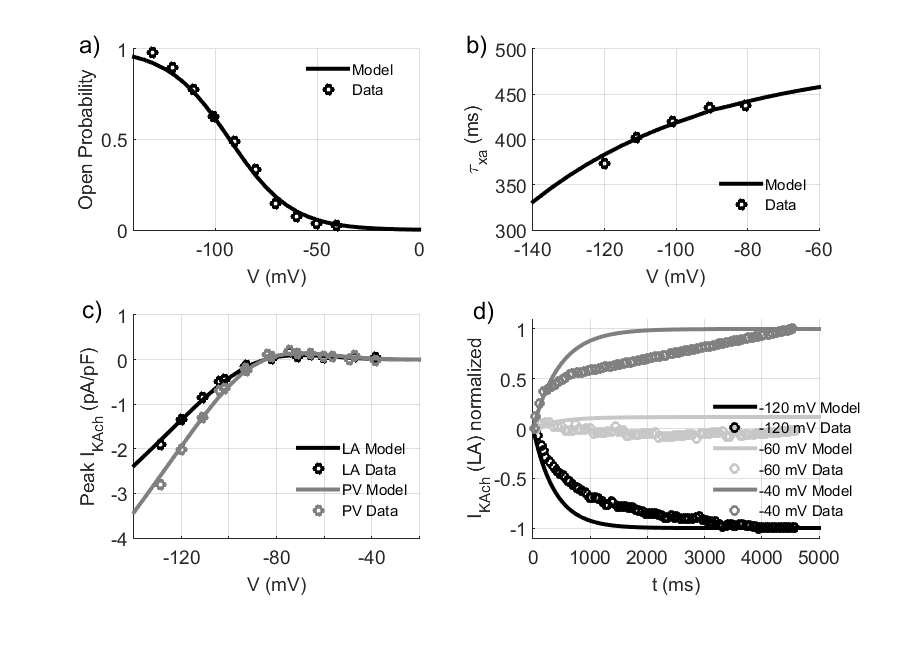

Supplement: S5 Fig — a) Steady-state values of the voltage-dependent activation variable (xa) as a function of membrane potential. b) Time constant of the voltage-dependent activation (τxa) variable as a function of membrane potential. c) Peak current-voltage relationship. d) Normalised current in the LA model as a function of time for voltage steps of -100, -40 and -20 mV (from -40 mV) as a function of time. (All experimental data for both LA and PV cells is taken from Ehrlich et al., 2004.) (PNG) [file pcbi.1005245.s006.png]

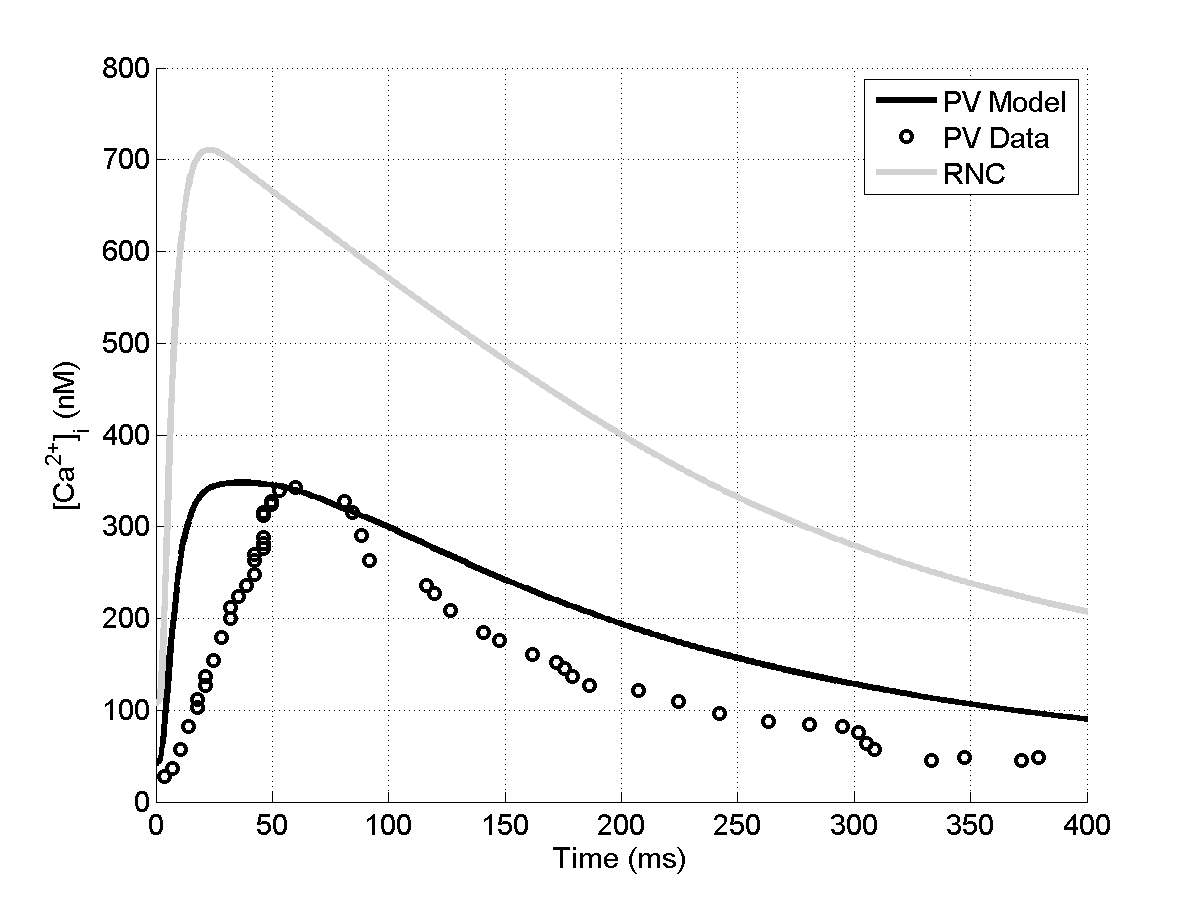

Supplement: S6 Fig — CaT in the PV model at 1Hz and comparison with RNC model and experimental data from Coutu et al., 2006. (PNG) [file pcbi.1005245.s007.png]

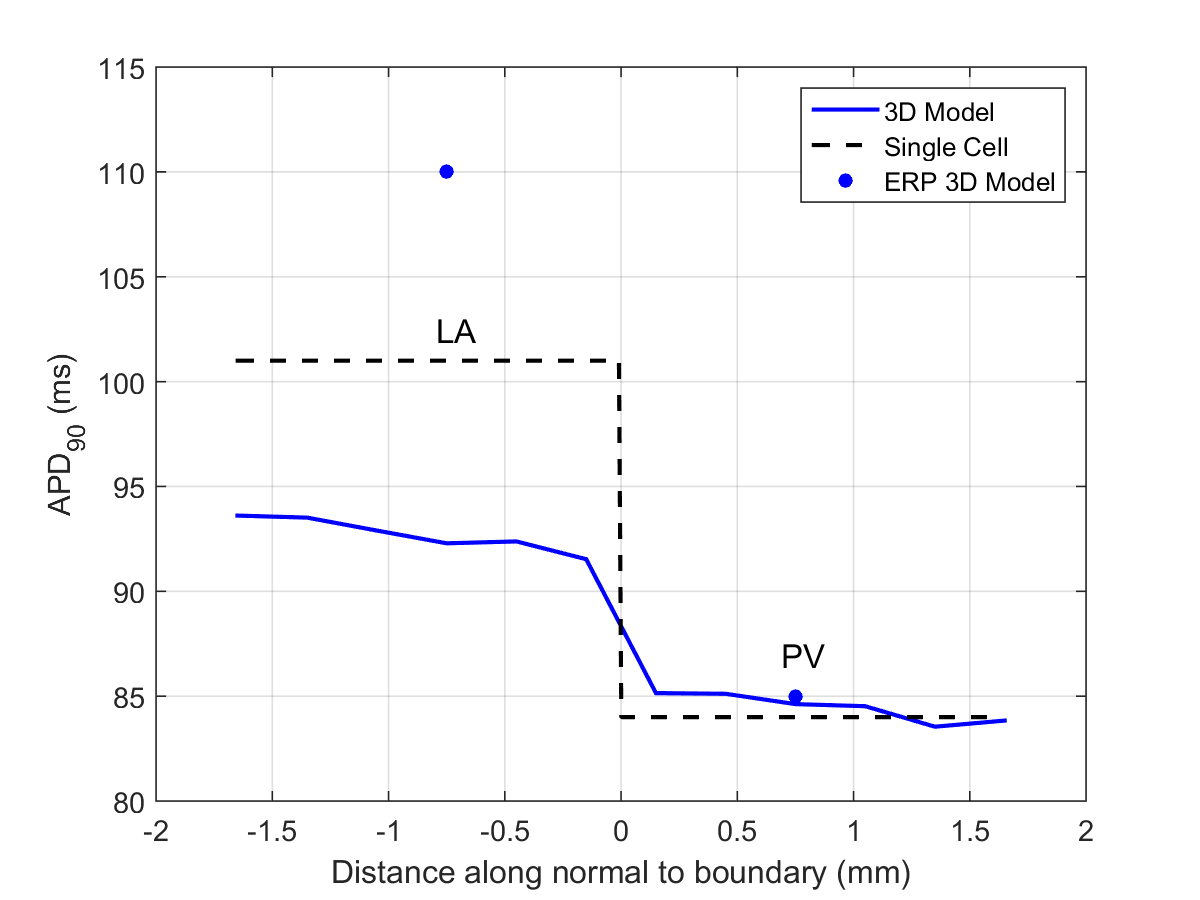

Supplement: S7 Fig — APD90 was measured across the LA-PV boundary in 3D (full blue line) and compared against single-cell APD90 values for corresponding cell types (dashed black line). ERPs (measured in 3D) for each of the tissue types are also shown (dark blue circles). Simulations were carried out at a BCL of 300 ms, with moderate ionic remodelling and CV reduction conditions. (PNG) [file pcbi.1005245.s008.png]

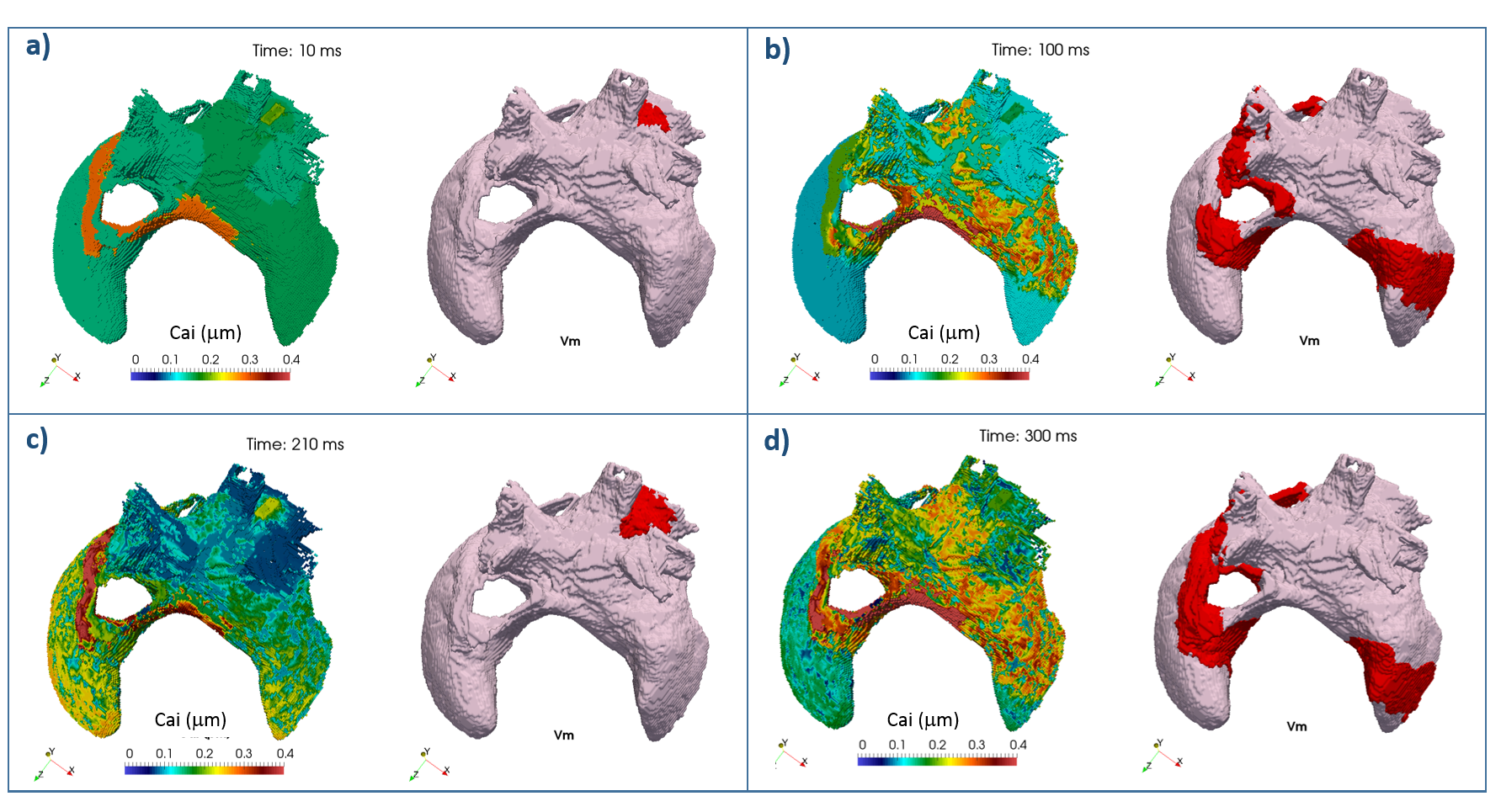

Supplement: S8 Fig — Pacing was performed in the left superior PV, in conditions of CV reduction and moderate ionic remodelling. Impulses are applied at 0 and 200 ms. The depolarizing wavefront (V > - 20mV) can be seen in red on the right hand side of each panel for the same time points as the Cai maps on the left hand side. Time since initial pacing: a) 10 ms, b) 100 ms, c) 210 ms, d) 300 ms. (PNG) [file pcbi.1005245.s009.png]

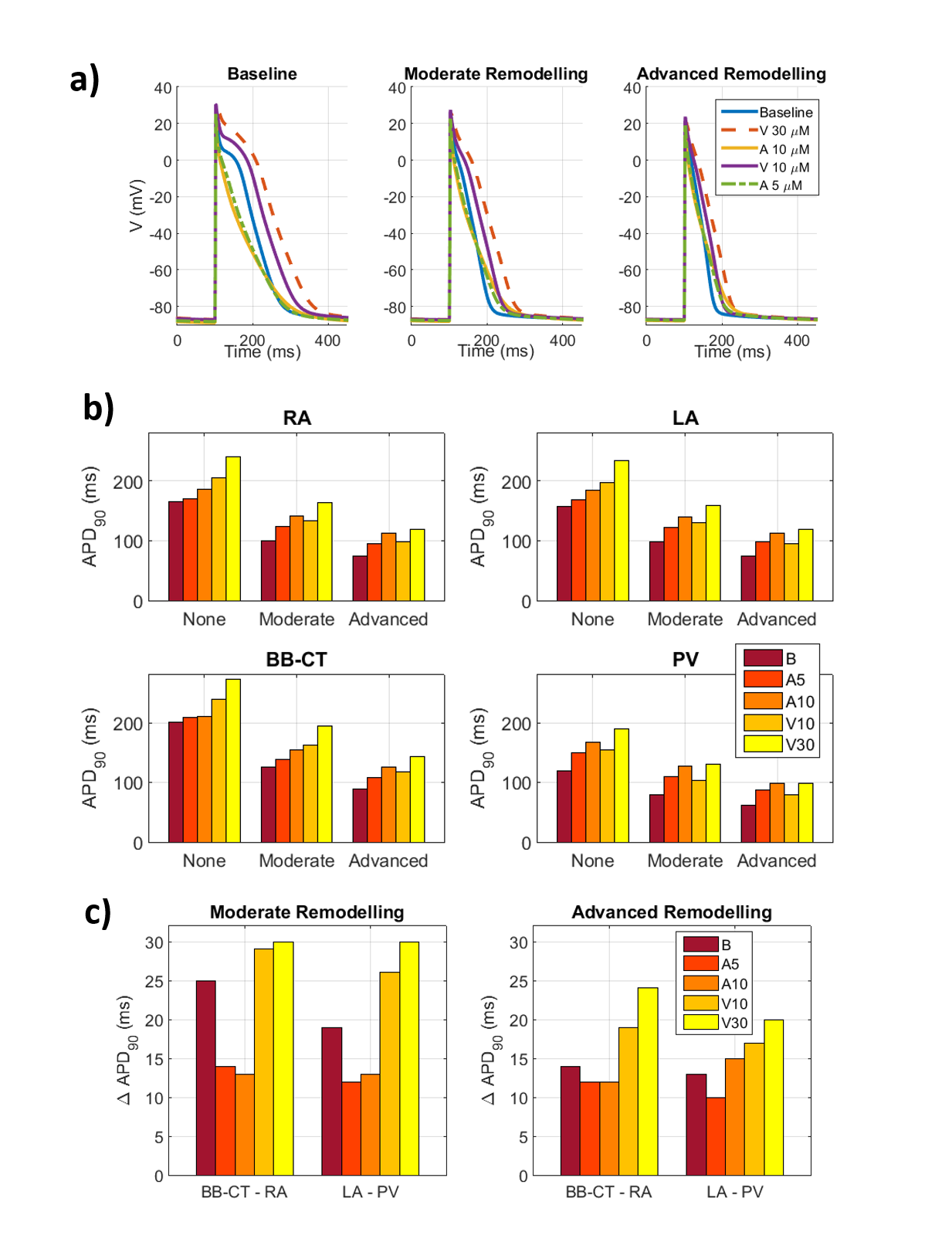

Supplement: S9 Fig — a) Action potentials for the right atrium, paced at 2 Hz, in the absence of drugs and after administration of vernakalant or amiodarone for different degrees of ionic remodelling. b) Absolute APD90 values in all cell types and c) differences in APD90 between right atrial tissues (BB-CT and RA) and left atrial tissues (PV and LA) for: different degrees of remodelling (none, moderate or advanced) at baseline (B) and after the application of either 5, 10 μM of amiodarone (A5, A10) or 10, 30 μM of vernakalant (V10, V30). (PNG) [file pcbi.1005245.s010.png]

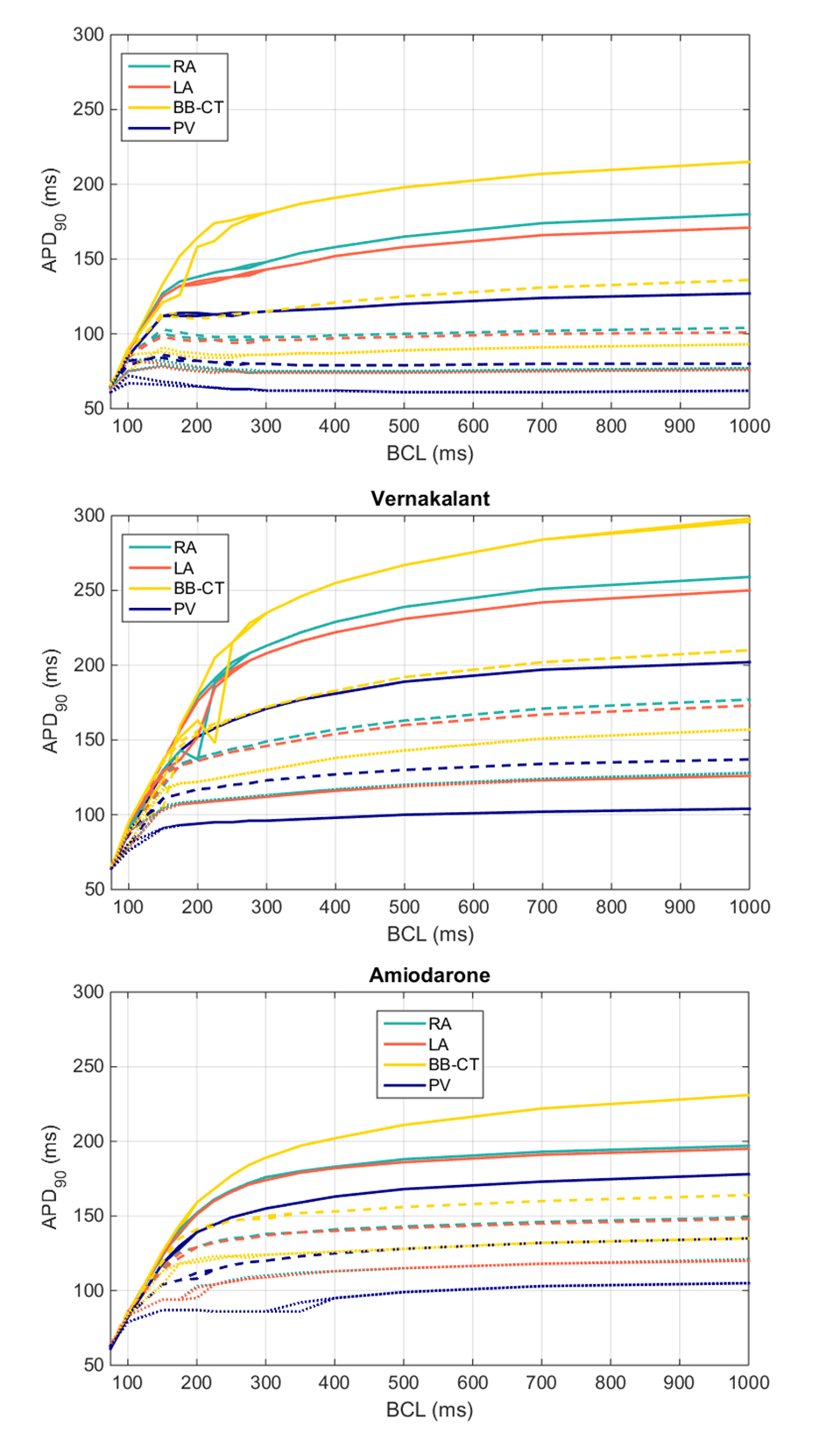

Supplement: S10 Fig — APD90 restitution curves in baseline (full line), moderate (dashed line) and advanced (dotted line) ionic remodelling for all atrial cell types in baseline conditions (top panel) and after the administration of 30 μM of vernakalant (centre panel) or 10 μM amiodarone (bottom panel). (PNG) [file pcbi.1005245.s011.png]

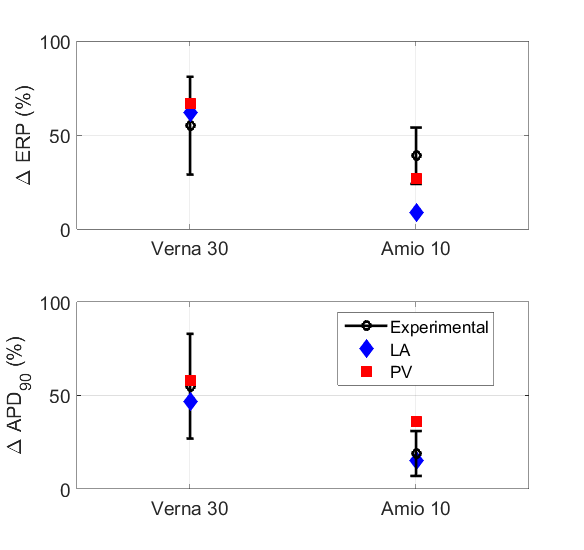

Supplement: S11 Fig — Experimental data (black) is taken from Sicouri et al., 2012 for the action of vernakalant 30 μM on healthy canine PVs and Sicouri et al., 2010 for the action of chronic amiodarone on healthy canine RA cells. Simulations were carried out for the PV and LA cells using the described 1D cable model (for ERP calculations) and single-cell models (for APD90) at a BCL of 500 ms in the absence of any remodelling. (PNG) [file pcbi.1005245.s012.png]

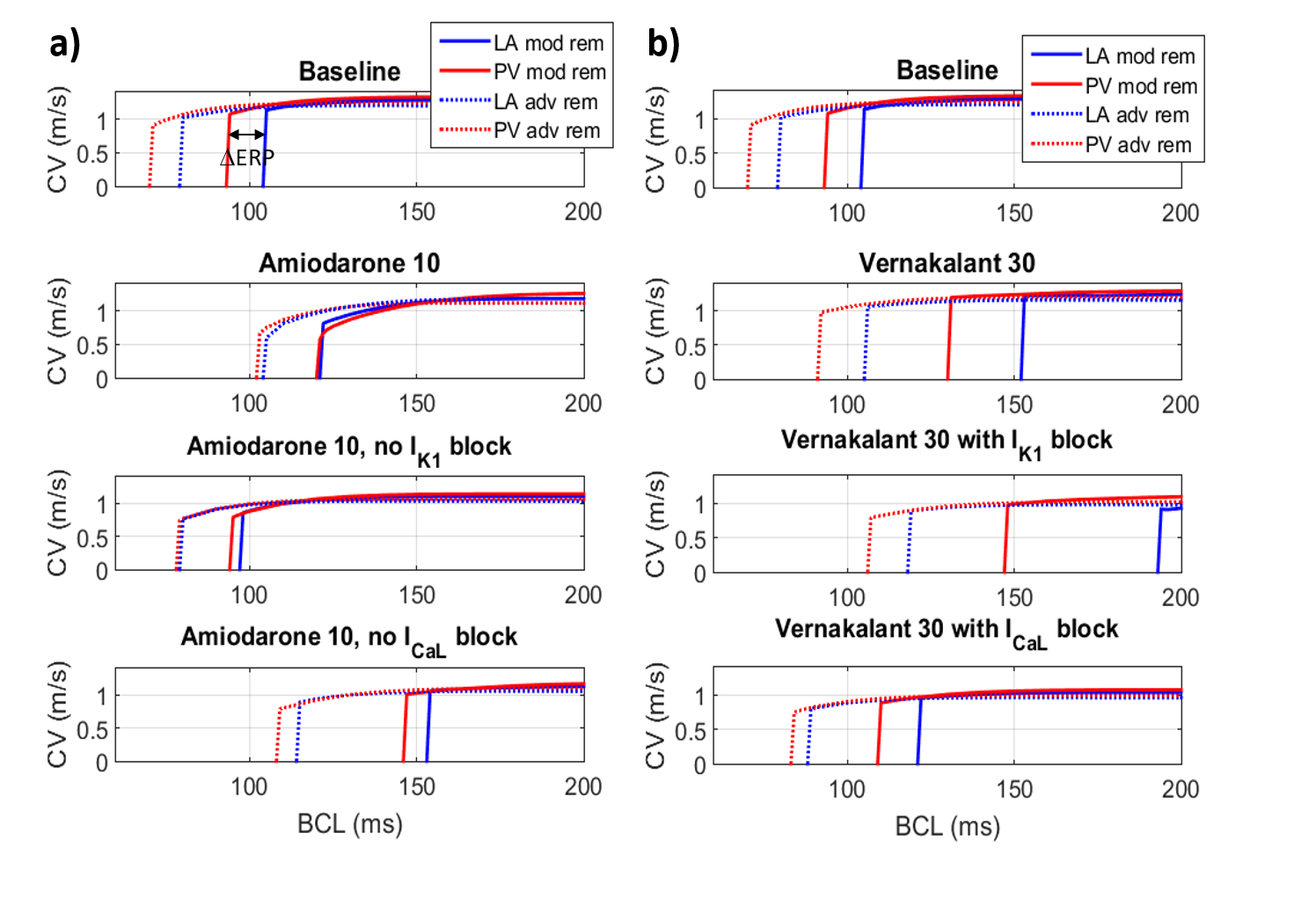

Supplement: S12 Fig — Conduction velocity restitution curves simulated in 1D for PV (red) and LA (blue) cells, in conditions of moderate (full lines) and severe remodelling (dashed lines). Panels show CV restitution curves for baseline values (top), in the presence of amiodarone 10 μM (a), second panel) and vernakalant 30 μM (b), second panel). The bottom two panels depict CV restitution curves for single-ionic channel modifications of amiodarone 10 μM (a)) or vernakalant 30 μM (b)). (PNG) [file pcbi.1005245.s013.png]

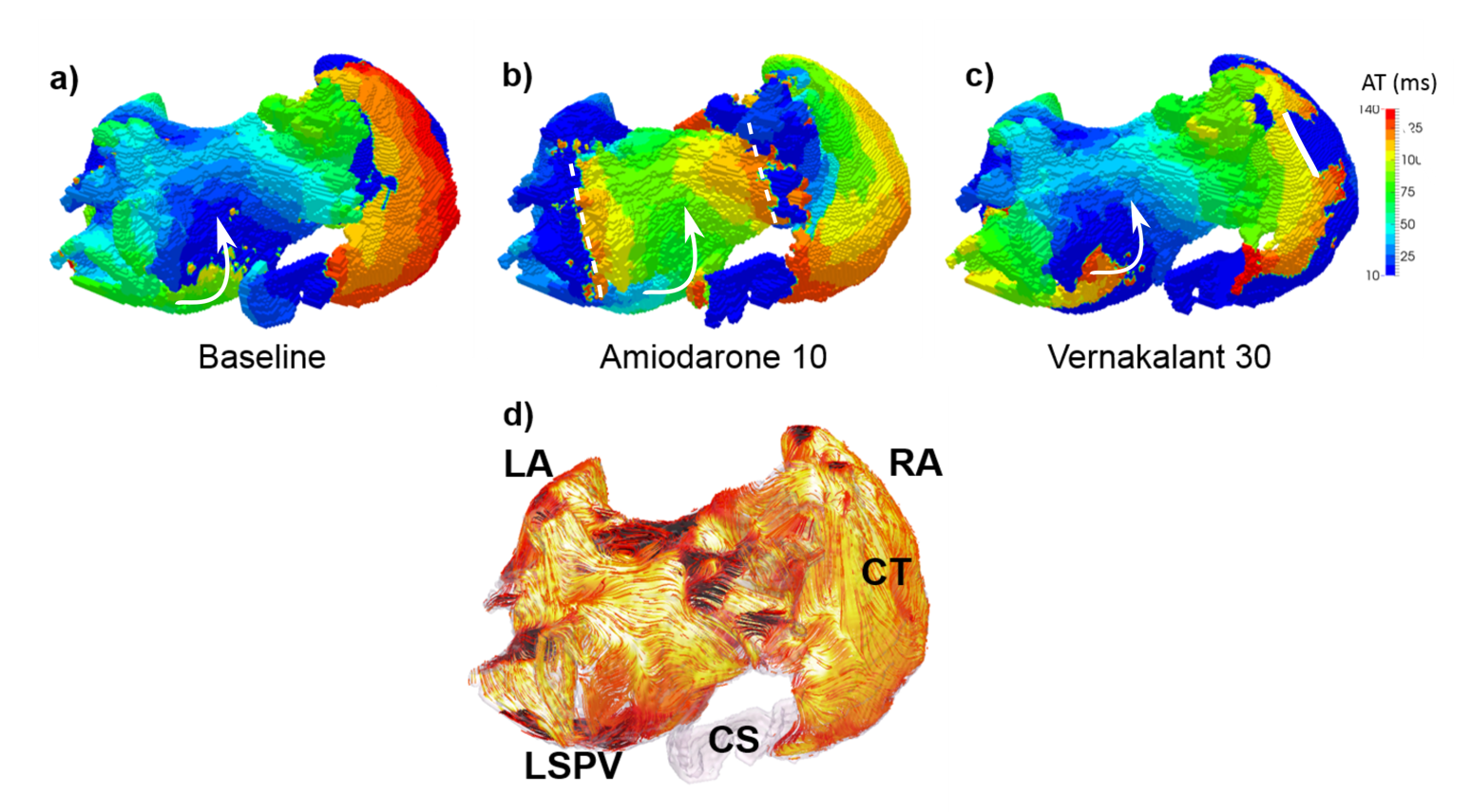

Supplement: S13 Fig — Superior-posterior view of the atria, showing ATs during AF (moderate ionic remodelling, CV reduction conditions) at a) baseline and after the application of b) amiodarone 10 μM and c) vernakalant 30 μM. Panel d) shows the 3D fibre orientation in the same geometry. The rotor around the LSPV (arrow) is terminated near the PVs by amiodarone (dashed lines), whereas vernakalant creates an additional conduction block at the CT (full line). (PNG) [file pcbi.1005245.s014.png]
