# Supplementary material for: Atrial Heterogeneity Generates Re-entrant Substrate during Atrial Fibrillation and Anti-arrhythmic Drug Action: Mechanistic Insights from Canine Atrial Models
Source: PLoS Comput Biol. 2016 Dec 16;12(12):e1005245. doi: 10.1371/journal.pcbi.1005245 (PMC5161306; doi:10.1371/journal.pcbi.1005245)
Supplement: S1 Table — *The formulation of these currents is also cell-type dependent—see the last chapter of S1 Supporting Text for details. (PDF) [file pcbi.1005245.s015.pdf]

**Conductance (nS/pF)**	**PV**	**LA**	**RA**	**BB - CT**	**RNC**
**gNa**	7.8	7.8	7.8	7.8	7.8
**gCaL**	0.255	0.34	0.34	0.58	0.24
**gCaP**	0.275	0.275	0.275	0.275	0.275
**gK1***	0.036	0.1	0.1	0.1	0.15
**gto**	0.07104	0.096	0.096	0.096	0.19824
**gKur**	0.0115	0.0115	0.0115	0.0115	1
**gbCl**	0.0055	0.0008	0.0008	0.0008	0
**gKr***	0.022475	0.0145	0.00899	0.00899	0.06984
**gKs**	0.0832	0.052	0.052	0.052	0.0561
**gbNa**	1.00E-05	1.00E-05	1.00E-05	1.00E-05	6.74E-04
**gbCa**	1.00E-05	1.00E-05	1.00E-05	1.00E-05	1.13E-03
**gNaCa**	1600	1600	1600	1600	1600
**gNaK**	0.6	0.6	0.6	0.6	0.6
**gKAch**	0.0065	0.0045	0.0045	0.0045	0

Table S1: Conductances of the currents used in each regional model. *The formulation of these currents is also cell-type dependent - see the last chapter of S1 Supporting Text for details.
