# Supplementary material for: Atrial Heterogeneity Generates Re-entrant Substrate during Atrial Fibrillation and Anti-arrhythmic Drug Action: Mechanistic Insights from Canine Atrial Models
Source: PLoS Comput Biol. 2016 Dec 16;12(12):e1005245. doi: 10.1371/journal.pcbi.1005245 (PMC5161306; doi:10.1371/journal.pcbi.1005245)
Supplement: S2 Table — Only parameters whose values differ in the two models are listed. (PDF) [file pcbi.1005245.s016.pdf]

**Variable**	**RNC Model**	**RA, LA, BB-CT, PV**	**Units**
k_{up}	9.2×10^{-4}	6.0×10^{-4}	μM
$i_{up,max}$	5.0×10^{-3}	3.5×10^{-3}	mM/ms
$ca_{up,max}$	15.0	27.0	mM
g_{rel}	30.0	8.0	kHz
τ_u	8.0	11.2	ms

Table S2: Ca^{2+}-handling variables in the proposed family of models and the RNC model. Only parameters whose values differ in the two models are listed.
