# Supplementary material for: Atrial Heterogeneity Generates Re-entrant Substrate during Atrial Fibrillation and Anti-arrhythmic Drug Action: Mechanistic Insights from Canine Atrial Models
Source: PLoS Comput Biol. 2016 Dec 16;12(12):e1005245. doi: 10.1371/journal.pcbi.1005245 (PMC5161306; doi:10.1371/journal.pcbi.1005245)
Supplement: S3 Table — (PDF) [file pcbi.1005245.s017.pdf]

Source of canine experimental data	Symbol in Figure 1a-c	Symbol in Figure 1d	AP data in Figure 3
**Ehrlich et al, 2003 [1]**	◊	△	
**Li et al, 2001 [2]**	O	□	LA (panel a)
**Cha et al, 2005 [3]**	□	X	LA, PV (panels a,b)
**Yue et al, 1997 [4]**	X	O	
**Burashnikov et al, 2004 [5]**		$<$	RA, BB-CT (panels c,d)
**Chen et al, 2006 [6]**		$>$	

Table S3: Literature references for the canine experimental data shown in **Figures 1, 3** and **4** in the manuscript.

References

[1] Ehrlich, J.R., Cha, T.J., Zhang, L., Chartier, D., Melnyk, P., Hohnloser, S.H., Nattel, S.: Cellular electrophysiology of canine pulmonary vein cardiomyocytes: action potential and ionic current properties. J. Physiol. **551** (sep 2003) 801–13

[2] Li, D.K.B., Zhang, L., Kneller, J., Nattel, S.: Potential Ionic Mechanism for Repolarization Differences Between Canine Right and Left Atrium. Circ. Res. **88**(11) (may 2001) 1168–1175

[3] Cha, T.J., Ehrlich, J.R., Zhang, L., Chartier, D., Leung, T.K., Nattel, S.: Atrial tachycardia remodeling of pulmonary vein cardiomyocytes: comparison with left atrium and potential relation to arrhythmogenesis. Circulation **111**(6) (feb 2005) 728–35

[4] Yue, L., Feng, J., Gaspo, R., Li, G.R., Wang, Z., Nattel, S.: Ionic Remodeling Underlying Action Potential Changes in a Canine Model of Atrial Fibrillation. Circ. Res. **81**(4) (oct 1997) 512–525

[5] Burashnikov, A., Mannava, S., Antzelevitch, C.: Transmembrane action potential heterogeneity in the canine isolated arterially perfused right atrium: effect of IKr and IKur/Ito block. Am. J. Physiol. Heart Circ. Physiol. **286**(6) (jun 2004) H2393–400

[6] Chen, Y.J., Chen, Y.C., Tai, C.T., Yeh, H.I., Lin, C.I., Chen, S.A.: Angiotensin II and angiotensin II receptor blocker modulate the arrhythmogenic activity of pulmonary veins. Br. J. Pharmacol. **147**(1) (jan 2006) 12–22
