# Supplementary material for: Atrial Heterogeneity Generates Re-entrant Substrate during Atrial Fibrillation and Anti-arrhythmic Drug Action: Mechanistic Insights from Canine Atrial Models
Source: PLoS Comput Biol. 2016 Dec 16;12(12):e1005245. doi: 10.1371/journal.pcbi.1005245 (PMC5161306; doi:10.1371/journal.pcbi.1005245)
Supplement: S4 Table — These are shown in conditions of moderate (7-days of RAP) and severe (42-days of RAP) remodelling, accompanied by the relevant references. (PDF) [file pcbi.1005245.s018.pdf]

Current	Moderate Remodelling	Severe Remodelling	References
I_{Na}	0.90	0.80	[1]
I_{CaL}	0.47	0.31	[2, 3]
I_{K1}	1.80	2.57	[3]
I_{KAch}	0.54	0.38	[4]
I_{to}	1.64	2.34	[2, 3]
I_{NaCa}	1.8	2.57	[5]

Table S4: Multiplicative factors for ionic remodelling of each current in conditions of moderate (7-days of RAP) and severe (42-days of RAP) remodelling, accompanied by the relevant references.

References

[1] Gaspo, R., Bosch, R.F., Bou-Abboud, E., Nattel, S.: Tachycardia-Induced Changes in Na+ Current in a Chronic Dog Model of Atrial Fibrillation. Circ. Res. **81**(6) (dec 1997) 1045–1052

[2] Yue, L., Feng, J., Gaspo, R., Li, G.R., Wang, Z., Nattel, S.: Ionic Remodeling Underlying Action Potential Changes in a Canine Model of Atrial Fibrillation. Circ. Res. **81**(4) (oct 1997) 512–525

[3] Cha, T.J., Ehrlich, J.R., Zhang, L., Chartier, D., Leung, T.K., Nattel, S.: Atrial tachycardia remodeling of pulmonary vein cardiomyocytes: comparison with left atrium and potential relation to arrhythmogenesis. Circulation **111**(6) (feb 2005) 728–35

[4] Voigt, N., Maguy, A., Yeh, Y., Qi, X.Y., Ravens, U., Dobrev, D., Nattel, S.: Changes in I K, ACh single-channel activity with atrial tachycardia remodelling in canine atrial cardiomyocytes. Cardiovasc. Res. **77**(1) (jan 2008) 35–43

[5] Cha, T.J., Ehrlich, J.R., Zhang, L., Nattel, S.: Atrial ionic remodeling induced by atrial tachycardia in the presence of congestive heart failure. Circulation **110**(12) (sep 2004) 1520–6
