# Supplementary material for: Atrial Heterogeneity Generates Re-entrant Substrate during Atrial Fibrillation and Anti-arrhythmic Drug Action: Mechanistic Insights from Canine Atrial Models
Source: PLoS Comput Biol. 2016 Dec 16;12(12):e1005245. doi: 10.1371/journal.pcbi.1005245 (PMC5161306; doi:10.1371/journal.pcbi.1005245)
Supplement: S5 Table — These are shown for three different stages of structural remodelling: baseline, CV reduction and additional AR increase. (PDF) [file pcbi.1005245.s019.pdf]

$D_{long}(mm^2/ms); AR$	**RA**	**LA**	**BB-CT**	**PV**
**Baseline**	0.4; 10	0.4; 10	0.6; 16	0.4; 16
**CV Reduction**	0.2; 10	0.2; 10	0.4; 16	0.2; 16
**AR Increase**	0.2; 16	0.2; 22	0.4; 16	0.2; 22

Table S5: Values for the longitudinal diffusion coefficient (D_{long}) and anisotropy ratio (AR) for each region of the atrial at three different stages of structural remodelling: baseline, CV reduction and additional AR increase.
