# Supplementary material for: Atrial Heterogeneity Generates Re-entrant Substrate during Atrial Fibrillation and Anti-arrhythmic Drug Action: Mechanistic Insights from Canine Atrial Models
Source: PLoS Comput Biol. 2016 Dec 16;12(12):e1005245. doi: 10.1371/journal.pcbi.1005245 (PMC5161306; doi:10.1371/journal.pcbi.1005245)
Supplement: S6 Table — Values from the current study computed at a BCL of 350 ms are shown alongside corresponding experimental values measured experimentally in dogs. (PDF) [file pcbi.1005245.s020.pdf]

$CV_{longitudinal}$ **(m/s)**	**Model**	**Canine Experiments**
**RA, no remodelling**	0.94	0.80-1.10 [1, 2, 3, 4]
**BB, no remodelling**	1.18	1.00-1.30 [2, 5, 6, 7]
**RA, after remodelling/RAP**	0.64	0.60-0.90 [2, 1, 8]

Table S6: Longitudinal conduction velocity values ($CV_{longitudinal}$) measured in the 3D atrial model in the current study at a BCL of 350 ms, alongside corresponding experimental values measured experimentally in dogs.

References

[1] Gaspo, R., Bosch, R.F., Talajic, M., Nattel, S.: Functional Mechanisms Underlying Tachycardia-Induced Sustained Atrial Fibrillation in a Chronic Dog Model. Circulation **96**(11) (dec 1997) 4027–4035

[2] Li, D.K.B., Fareh, S., Leung, T.K., Nattel, S.: Promotion of Atrial Fibrillation by Heart Failure in Dogs : Atrial Remodeling of a Different Sort. Circulation **100**(1) (jul 1999) 87–95

[3] Guerra, J.M.: Effects of the Gap Junction Modifier Rotigaptide (ZP123) on Atrial Conduction and Vulnerability to Atrial Fibrillation. Circulation **114**(2) (jul 2006) 110–118

[4] Koura, T.: Anisotropic Conduction Properties in Canine Atria Analyzed by High-Resolution Optical Mapping: Preferential Direction of Conduction Block Changes From Longitudinal to Transverse With Increasing Age. Circulation **105**(17) (apr 2002) 2092–2098

[5] Spach, M.S., Miller, W.T., Dolber, P.C., Kootsey, J.M., Sommer, J.R., Mosher, C.E.: The functional role of structural complexities in the propagation of depolarization in the atrium of the dog. Cardiac conduction disturbances due to discontinuities of effective axial resistivity. Circ. Res. **50**(2) (feb 1982) 175–191

[6] Rensma, P.L., Allessie, M.A., Lammers, W.J., Bonke, F.I., Schalij, M.: Length of excitation wave and susceptibility to reentrant atrial arrhythmias in normal conscious dogs. Circ. Res. **62**(2) (feb 1988) 395–410

[7] Dolber, P.C., Spach, M.S.: Structure of canine Bachmann's bundle related to propagation of excitation. Am J Physiol Hear. Circ Physiol **257**(5) (nov 1989) H1446–1457

[8] Kumagai, K., Nakashima, H., Urata, H., Gondo, N., Arakawa, K., Saku, K.: Effects of angiotensin II type 1 receptor antagonist on electrical and structural remodeling in atrial fibrillation. J. Am. Coll. Cardiol. **41**(12) (jun 2003) 2197–2204
