# Supplementary material for: Atrial Heterogeneity Generates Re-entrant Substrate during Atrial Fibrillation and Anti-arrhythmic Drug Action: Mechanistic Insights from Canine Atrial Models
Source: PLoS Comput Biol. 2016 Dec 16;12(12):e1005245. doi: 10.1371/journal.pcbi.1005245 (PMC5161306; doi:10.1371/journal.pcbi.1005245)
Supplement: S7 Table — SVC: superior vena cava; RAA: right atrial appendage; IVC: inferior vena cava; LBB: left portion of Bachmann's bundle; LAA: left atrial appendage; RSPV: right superior pulmonary vein; RIPV: right inferior pulmonary vein. (PDF) [file pcbi.1005245.s021.pdf]

Region	Sakamoto et al, 2005 [1]	Hayashi et al, 1982 [2]	Model
**SVC**	15 ms	10 ms	14 ms
**RAA**	25 ms	37 ms	38 ms
**IVC**	45 ms	35 ms	40 ms
**LBB**	35 ms	25 ms	28 ms
**LAA**	65 ms	55 ms	68 ms
**RSPV**	50 ms	40 ms	39 ms
**RIPV**	55 ms	30 ms	49 ms

Table S7: Activation times in different regions when pacing from the sino-atrial node, at a BCL of 350 ms. SVC: superior vena cava; RAA: right atrial appendage; IVC: inferior vena cava; LBB: left portion of Bachmann's bundle; LAA: left atrial appendage; RSPV: right superior pulmonary vein; RIPV: right inferior pulmonary vein.

References

[1] Sakamoto, S.I., Nitta, T., Ishii, Y., Miyagi, Y., Ohmori, H., Shimizu, K.: Interatrial electrical connections: the precise location and preferential conduction. J. Cardiovasc. Electrophysiol. **16**(10) (oct 2005) 1077–86

[2] Hayashi, H., Lux, R.L., Wyatt, R.F., Burgess, M.J., Abildskov, J.A.: Relation of canine atrial activation sequence to anatomic landmarks. Am. J. Physiol. **242**(3) (mar 1982) H421–H428
