# Supplementary material for: Atrial Heterogeneity Generates Re-entrant Substrate during Atrial Fibrillation and Anti-arrhythmic Drug Action: Mechanistic Insights from Canine Atrial Models
Source: PLoS Comput Biol. 2016 Dec 16;12(12):e1005245. doi: 10.1371/journal.pcbi.1005245 (PMC5161306; doi:10.1371/journal.pcbi.1005245)
Supplement: S8 Table — The values used to model 5 and 10 μM amiodarone (Amio) and 10 and 30 μM vernakalant (Verna) are accompanied by IC50 values from the literature for each current affected by the drug. Corresponding literature references are also shown. (PDF) [file pcbi.1005245.s022.pdf]

**Current**	**Amio 5**μM	**Amio 10** μM	IC_{50} **Amio** (μM)	**Verna 10** μM	**Verna 30** μM	IC_{50} **Verna** (μM)
I_{Na}	0.90	0.81	4.8 [1], > 3 [2]	1.00	0.90	107 ± 11 [3]
I_{CaL}	0.50	0.35	10.0 [4], 5.8 [5]	1	1	-
I_{Ks}	0.50	0.35	3.8 [6], 0.1 [2]	1	1	-
I_{Kr}	0.50	0.35	2.8 [7], 9.8 [8]	0.68	0.41	21 ± 3 [3]
I_{K1}	0.70	0.49	30 [2], > 15 [9]	1	1	-
I_{to}	1	1	-	0.56	0.30	15 ± 2 [3]
I_{Kur}	1	1	-	0.55	0.30	13 ± 1 [3]
I_{KAch}	0.22	0.15	1.0 [2]	0.54	0.22	10 ± 1 [3]

Table S8: Blockade factors for 5 and 10 μM amiodarone (Amio) and vernakalant (Verna) at 10 and 30 μM, accompanied by IC_{50} values from the literature for each current affected by the drug. Corresponding literature references are also shown.

References

[1] Lalevée, N., Barrère-lemaire, S., Gautier, P., Nargeot, J., Richard, S.: Effects of Amiodarone and Dronedarone on Voltage-Dependent Sodium Current in Human Cardiomyocytes. J. Cardiovasc. Electrophysiol. **14**(8) (aug 2003) 885–890

[2] Patel, C., Yan, G.X., Kowey, P.R.: Dronedarone. Circulation **120**(7) (aug 2009) 636–44

[3] Fedida, D.: Vernakalant (RSD1235): a novel, atrial-selective antifibrillatory agent. Expert Opin. Investig. Drugs **16**(4) (apr 2007) 519–532

[4] Hancox, J.C.: Amiodarone blocks L-type calcium current in single myocytes isolated from the rabbit atrioventricular node. Gen. Pharmacol. Vasc. Syst. **29**(3) (sep 1997) 429–435

[5] Nishimura, M., Follmer, C., Singer, D.: Amiodarone blocks calcium current in single guinea pig ventricular myocytes. J. Pharmacol. Exp. Ther. **251**(2) (nov 1989) 650–659

[6] Loewe, A., Lutz, Y., Wilhelms, M., Sinnecker, D., Barthel, P., Scholz, E., Doessel, O., Schmidt, G., Seemann, G.: In-silico assessment of the dynamic effects of amiodarone and dronedarone on human atrial patho-electrophysiology. Europace **16 Suppl 4**(suppl_4) (nov 2014) iv30–iv38

[7] Kamiya, K., Nishiyama, A., Yasui, K., Hojo, M., Sanguinetti, M.C., Kodama, I.: Short- and Long-Term Effects of Amiodarone on the Two Components of Cardiac Delayed Rectifier K+ Current. Circulation **103**(9) (mar 2001) 1317–1324

[8] Kiehn, J., Thomas, D.L., Karle, C.A., Schöls, W., Kübler, W.: Inhibitory effects of the class III antiarrhythmic drug amiodarone on cloned HERG potassium channels. Naunyn. Schmiedebergs. Arch. Pharmacol. **359**(3) (mar 1999) 212–9

[9] Sato, R., Koumi, S., Singer, D., Hisatome, I., Jia, H., Eager, S., Wasserstrom, J.: Amiodarone blocks the inward rectifier potassium channel in isolated guinea pig ventricular cells. J. Pharmacol. Exp. Ther. **269**(3) (jun 1994) 1213–1219
