# Supplementary material for: Atrial Heterogeneity Generates Re-entrant Substrate during Atrial Fibrillation and Anti-arrhythmic Drug Action: Mechanistic Insights from Canine Atrial Models
Source: PLoS Comput Biol. 2016 Dec 16;12(12):e1005245. doi: 10.1371/journal.pcbi.1005245 (PMC5161306; doi:10.1371/journal.pcbi.1005245)
Supplement: S9 Table — Modified amiodarone 10 μM (Amio 10) and 30 μM vernakalant (Verna 30) drugs were applied and their outcome is compared to baseline (no drug) conditions. All simulations lasted 5 s. Blockade factors for additional IK1 and ICaL blocks were 0.60. (PDF) [file pcbi.1005245.s023.pdf]

**Ionic Remodel.**	**Baseline**	**Amio 10 - I_{K1} block**	**Amio 10 - I_{CaL} block**	**Verna 30 + I_{K1} block**	**Verna 30 + I_{CaL} block**
**Moderate**	PV: 2 rotors RA: wavelets	PV: 2 rotors RA: 1 rotor	All activity terminated	PV: 1 rotor RA: wavelets	PV: 1 rotor RA: wavelets
**Severe**	PV: 2 rotors LA: 1 rotor RA: 1 rotor, wavelets	PV: 2 rotors RA: 1 rotor, wavelets	All activity terminated	PV: 2 rotors RA: wavelets	PV: 2 rotors RA: 1 rotor

Figure S9: Outcome of 3D simulations after administration of modified amiodarone 10 (Amio 10) and vernakalant 30 (Verna 30) drugs, in comparison to baseline (no drug) conditions. All simulations lasted 5 s. Blockade factors for additional I_{K1} and I_{CaL} blocks were 0.60.
