# Supplementary material for: Atrial Heterogeneity Generates Re-entrant Substrate during Atrial Fibrillation and Anti-arrhythmic Drug Action: Mechanistic Insights from Canine Atrial Models
Source: PLoS Comput Biol. 2016 Dec 16;12(12):e1005245. doi: 10.1371/journal.pcbi.1005245 (PMC5161306; doi:10.1371/journal.pcbi.1005245)
Supplement: S10 Table — We show the changes in effective refractory period (ERP) and dispersion of ERP between the LA and PV cells effected by the analysed drug actions (shown in S12 Fig), as well as the qualitative outcome of 3D simulations (S9 Table) for the same drug actions. (PDF) [file pcbi.1005245.s024.pdf]

	Amiodarone 10	Amiodarone 10, no I_{K1} block	Amiodarone 10, no I_{CaL} block
**ERP**	increased	reduced	increased
**Δ ERP**	reduced	reduced	reduced
**3D outcome**	good	poor	good

	Vernakalant 30	Vernakalant 30, with I_{K1} block	Vernakalant 30, with I_{CaL} block
**ERP**	increased	increased	increased
**Δ ERP**	increased	increased	increased
**3D outcome**	poor	poor	poor

Table S10: Summary of changes in effective refractory period (ERP) and dispersion of ERP between the LA and PV cells effected by analysed drug actions (shown in **Figure S12**), as well as the qualitative outcome of 3D simulations (**Table S9**) for the same drug actions.
